# Gabapentin in procedure-specific postoperative pain management – preplanned subgroup analyses from a systematic review with meta-analyses and trial sequential analyses

**DOI:** 10.1186/s12871-017-0373-8

**Published:** 2017-06-21

**Authors:** Maria Louise Fabritius, Anja Geisler, Pernille Lykke Petersen, Jørn Wetterslev, Ole Mathiesen, Jørgen Berg Dahl

**Affiliations:** 1 Department of Anaesthesiology and Intensive Care, Bispebjerg and Frederiksberg Hospitals, Bispebjerg bakke 23, 2400 Copenhagen, NV Denmark; 2 Department of Anaesthesiology, Zealand University Hospital, Lykkebækvej 1, 4600 Køge, Denmark; 3 Department of Anaesthesiology, Centre of Head and Orthopaedics, Copenhagen University Hospital, Rigshospitalet, Blegdamsvej 9, 2100 Copenhagen, Denmark; 4 Copenhagen Trial Unit, Centre for Clinical Intervention Research, Copenhagen University Hospital, Rigshospitalet, Blegdamsvej 9, 2100 Copenhagen, Denmark; 5 Department of Anaesthesiology, Zealand University Hospital, Lykkebækvej 1, 4600 Køge, Denmark; 6 Department of Anaesthesiology and Intensive Care, Bispebjerg and Frederiksberg Hospitals, Bispebjerg bakke 23, 2400 Copenhagen, NV Denmark

**Keywords:** Gabapentin, Gamma-Aminobutyric acid, Analgesics, Therapeutic use, Pain, Drug therapy, Procedure-specific pain management, Postoperative pain management, Systematic review, Subgroup analyses

## Abstract

**Background:**

It has been argued that postoperative pain treatment should be “procedure-specific”, since different analgesics may have specific effects dependent on the surgical procedure. The aim of the present subgroup analysis was to compare the beneficial and harmful effects of perioperative gabapentin treatment in different surgical procedures.

**Methods:**

Relevant databases were searched for randomized clinical trials (RCTs) comparing gabapentin versus placebo. Two authors independently screened titles and abstracts, extracted data and assessed risk of bias. The primary outcomes were differences in 24-h morphine consumption, and serious adverse events (SAE) between surgical procedures. These subgroup analyses were predefined in a PRISMA compliant systematic review registered at PROSPERO (ID: CRD42013006538). It was predefined that conclusions should primarily be based on trials classified as overall low risk of bias.

**Results:**

Seventy-four RCTs with 5645 patients were included, assessing benefit and harm in cholecystectomy, hysterectomy, mastectomy, and arthroplasty surgery, spinal surgery, and thoracic surgery.

Only eight of 74 trials were classified as overall low risk of bias limiting our ability to conclude on the estimates in most meta-analyses. The differences between surgical procedures in these trials were not statistically significant when tested for subgroup differences. Fifteen trials with 1377 patients reported a total of 59 SAEs, most of which were observed in the thoracic surgery group.

**Conclusion:**

Both beneficial and harmful effects in these subgroup analyses were influenced by bias and insufficient data, limiting conclusions. With these limitations, we could not adequately test for differences in beneficial or harmful outcomes between six surgical subgroups undergoing perioperative gabapentin treatment.

**Electronic supplementary material:**

The online version of this article (doi:10.1186/s12871-017-0373-8) contains supplementary material, which is available to authorized users.

## Background

Pain management is a crucial component in postoperative care of the surgical patient. The combination of non-opioid and opioid analgesics, known as multimodal analgesia, is a cornerstone in the treatment of postoperative pain. Gabapentin has recently become a part of a wide array of postoperative multimodal analgesic regimens [1–3].

It has been argued that postoperative pain treatment should be “procedure-specific”, that is, adapted to the particular surgical procedure, since different analgesics may have specific effects dependent on the nature of the surgery [4, 5].

Gabapentin has been used in postoperative pain management since 2002. It is an anti-epileptic drug presumed to affect nociceptive processing through α2δ-subunits of voltage gated calcium channels, thereby causing decrease in excitatory neurotransmitters, e.g. glutamate, substance P and calcitonin gene-related peptide (CGRP) [6, 7]. The anti-hyperalgesic properties have been investigated in several experimental and clinical trials [8–11].

In a recent systematic review we pooled data from all clinical trials and different surgical interventions with gabapentin [12, 13]. The conclusion from this review was, that firm evidence for use of gabapentin is lacking, as clinically relevant beneficial effects of gabapentin may be absent and harm is imminent, especially when added to multimodal analgesic regimens [12]. In the present preplanned subgroup analyses and post hoc analyses, we aimed to compare the procedure-specific effects of peri-operative gabapentin on postoperative opioid consumption, pain intensity, and adverse- and serious adverse events in six different surgical procedures. It was our hypothesis that the reduction in 24-h morphine consumption and incidence of SAE’s would differ between surgical procedures.

## Methods

These are preplanned subgroup analyses and post hoc analyses from a systematic review following the methodology recommended by the Cochrane Collaboration. The protocol was published in the International Prospective Register of Systematic Reviews (PROSPERO) (www.crd.york.ac.uk/PROSPERO) registration no. CRD42013006538 [13].

### Search strategy

The search was planned by a trial search coordinator using the Cochrane Library’s CENTRAL, PubMed, EMBASE, and Science Citation Index Expanded databases. Previous reviews, reference lists and Google Scholar were hand-searched for eligible trials. Www.clinicaltrials.gov; www.controlled-trials.com; www.centerwatch.com; www.eudraCT.com, and at the homepage of the US Food and Drug Administration (FDA) were searched for unpublished trials. Non-English articles were translated to English. The electronic search (Additional file 1: Appendix 1 - search strategies) was last updated April 12th, 2016.

### Data extraction

After removal of duplicates, titles and abstracts were screened by two authors (MLF, AG). MLF and one other independent author (AG, MSH, PLP, LN) assessed full texts, extracted data and assessed bias. The following characteristics were extracted from the trials using a data extraction form: Year of publication, number of participants, type of surgery, follow-up period and dose regimen, consumption of opioid, and non-opioid medication, pain intensity, and any adverse events described in the trials, including serious adverse events (SAEs).

The corresponding author was contacted whenever data were insufficiently reported, and contact was repeated after 14 days. In case of no response, the involved bias domains were classified as unclear.

### Risk of bias assessment

Risk of bias was assessed using The Cochrane Handbook guidelines. All trials were classified as low, unclear or high risk of bias using the following domains: Random sequence generation, allocation concealment, blinding, incomplete outcome data, selective outcome reporting and other bias, including funding and confirmatory bias.

Disagreements between authors on study selection, data extraction or bias assessment were solved by OM, JBD or JW.

We decided a priori to report and conclude based primarily on results from trials classified as low risk of bias.

### Small trial size

All trials were evaluated in this post hoc analysis and allocated to the corresponding group according to the numbers of participants included in the analyses. Small trials were defined as trials with less than 50 patients included in each group. Trials were allocated to the remaining two groups if they included either more than 50 patients, or more than 200 patients [12].

### Analyses

The present subgroup analyses of surgical procedures were predefined in the protocol investigating the effect of different surgical procedures: Cholecystectomy, hysterectomy, mastectomy, orthopedic arthroplasty surgery, spinal and thoracic surgery on the primary and secondary outcomes. Analyses of thoracic surgery and orthopedic arthroplasty surgery have been added post hoc [13]. The surgical procedures were chosen to represent a wide range of surgical interventions and patient populations. Cholecystectomy is a minor procedure, often performed as day-case surgery, whereas thoracic surgery is a major procedure, which may be associated with intensive care in the immediate postoperative period. Mastectomy and hysterectomy are procedures with moderate to severe pain postoperatively. Orthopedic arthroplasty and spinal surgery often represents patients with chronic pain preoperatively. We chose to add trials investigating thoracic surgery and orthopedic arthroplasty surgery post hoc, in order to broaden the range of surgical interventions.

The planning and interpretation of the subgroup analyses followed the direction of the Cochrane Handbook [14].

### Outcomes

The primary outcomes were difference in 24-h postoperative opioid sparing effects, and reported serious adverse events (SAE) between surgical procedures. SAE’s were defined according to the International Conference of Harmonization – Good Clinical Practice (ICH-GCP) definitions: Medical events being either life threatening, resulting in death, disability or significant loss of function, or causing hospital admission or prolonged hospitalization [15].

Secondary outcomes were differences in early (6-h) and late (24-h) pain postoperatively, both at rest and during mobilization, and all other adverse events, between surgical procedures.

All opioids were converted to intravenous morphine based upon equivalency as presented in Additional file 1: Appendix 2. Various scales were used to report pain intensity in the trials. All pain intensity scales reporting pain levels between 0 and 10 were converted to the Visual Analogue Scale (VAS) 0 to 100 mm.

### Statistical analysis

Review Manager (RevMan) [Computer program], Version 5.1.6, Copenhagen: The Nordic Cochrane Centre, The Cochrane Collaboration, 2014, and Trial Sequential Analysis (TSA) software (version 0.9.5.5 Beta), Copenhagen Trial Unit, Denmark, was used for statistical analyses as predefined in the protocol.

In trials with more than one active treatment arm, including trials testing doses delivered pre- and immediate postoperatively, means and standard deviations were combined for the intervention groups.

Mean and standard deviations were estimated from median and range values according to the method described by Hozo et al. [16] Standard deviations were calculated by dividing the difference in interquartile ranges with 1.35 [14].

Longer ordinal scales were analyzed as continuous data. For dichotomous data, RR with a 95% confidence interval was calculated.

We examined the heterogeneity between trials using chi-squared test. The heterogeneity was measured by I^2^, which quantifies inconsistencies. If the I^2^ was greater than zero the results were calculated using both a fixed effect model (FEM) and random effect model (REM) and the most conservative estimation was used [14, 17]. In the case of very few and rare events, Peto’s odd ratio was used to provide the best coverage of confidence intervals [18, 19].

Estimates were pooled in meta-analyses whenever more than one trial was included for the outcome. Tests for subgroup differences were carried out for all surgical procedures on all outcomes whenever a meta-analysis was possible. Using RevMan, the method to test for subgroup differences was implemented for all types of meta-analyses [14]. Our test for subgroup differences was performed on each of the subgroups testing them for subgroup differences against the compiled, remaining subgroups using the chi-squared test.

We used Trial Sequential Analysis (TSA) in post hoc analyses to adjust the confidence intervals for sparse data and repetitive testing. Minimal clinical relevant differences were defined as in our main review [12]. In the event that the accrued information size was less than 5% of the required information size, no TSA was reported, as the TSA program is unable to calculate trial sequential monitoring boundaries in this situation.

## Results

**Fig. 1:**
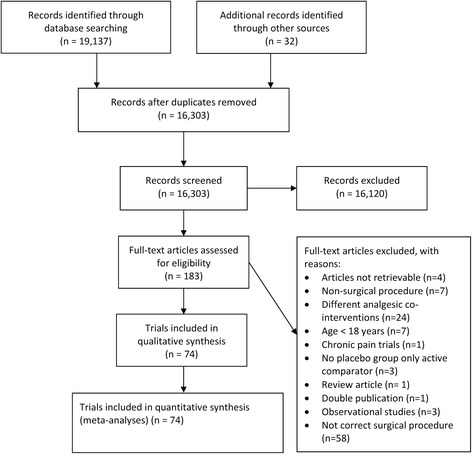
PRISMA flowchart

The search strategies revealed 19,137 titles. Duplicates were removed and 16,303 titles were sorted according to inclusion- and exclusion criteria. One-hundred-thirty-five randomized controlled trials and observational studies were included in the original systematic review. After excluding 61 trials investigating other surgical procedures, a total of 74 randomized controlled trials with 5645 patients were included in the present analyses (Fig. 1: PRISMA flowchart) [20–94].

### Characteristics of included trials

**Fig. 2:**
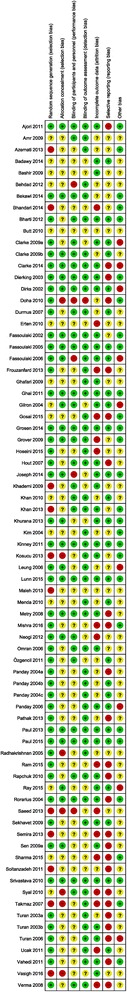
Risk of bias assessment. Risk of bias graph: The ‘Other’ bias domain is an evaluation of risk of financial bias and confirmatory bias

**Fig. 3:**
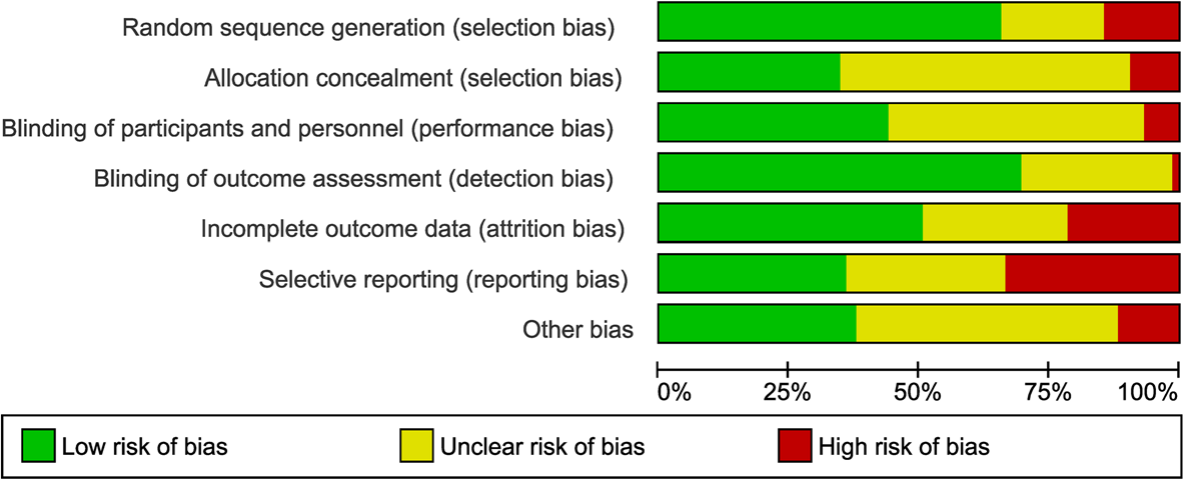
Risk of bias graph. Risk of bias summary

Trial characteristics are presented in Table 1. Eight trials were classified as overall low risk of bias, [39, 43, 46, 56, 59, 72, 73, 85] 18 trials were overall unclear risk of bias [21, 23, 24, 26, 28, 29, 31, 36, 38, 42, 52, 54, 55, 61, 65, 66, 68, 69, 80] and 48 trials were classified as high risk of bias, [20, 22, 25, 27, 30, 32–35, 37, 40, 41, 44, 45, 47–51, 53, 57, 58, 60, 62–64, 67, 70, 71, 74–79, 81–84, 86–94] (Fig. 2: Bias assessment). Allocation concealment, selective outcome reporting and “other bias” were the domains with most unclear or high risk of bias evaluations (Fig. 3: Risk of bias graph).

**Table 1:** Trial characteristics

Reference *(Author and year)*	Surgical procedure	N *Gabapentin/Control*	Intervention		Postoperative analgesia	Anesthetic technique	Bias assessment
			*Dose (mg)* *(mg/day* ^a^ *)*	*Single/Continuous*			
Cholecystectomy trials							
Bashir 2009 [24]	Laparoscopic cholecystectomy	50/50	600 mg	Single	Not described	GA	Unclear
Bekawi 2014 [26]	Laparoscopic cholecystectomy	30/30	1200 mg (400 mg)	Continuous	NSAID Pethidine Tramadol	GA	Unclear
Bhandari 2014 [27]	Laparoscopic cholecystectomy	20/20	600 mg (600 mg)	Continuous	NSAID	GA	High
Hosseini 2015 [48]	Laparoscopic cholecystectomy	22/22	600 mg	Single	Morphine	GA	High
Khademi 2009 [51]	Open cholecystectomy	44/43	600 mg	Single	Pethidine	GA	High
Mishra 2016 [63]	Laparoscopic cholecystectomy	30/30	900 mg	Single	Tramadol	GA	High
Neogi 2012 [64]	Laparoscopic cholecystectomy	30/30	900 mg	Single	Tramadol	GA	High
Panday 2004b [68]	Laparoscopic cholecystectomy	153/153	300 mg	Single	Fentanyl	GA	Unclear
Panday 2006 [70]	Laparoscopic cholecystectomy	125/125	600 mg	Single	Fentanyl	GA	High
Pathak 2013 [71]	Open cholecystectomy	40/40	1200 mg	Single	Pethidine	GA	High
Saeed 2013 [79]	Laparoscopic cholecystectomy	50/50	600 mg	Single	Pethidine NSAID	GA	High
Semira 2013 [81]	Laparoscopic cholecystectomy	30/30	600 mg	Single	Not described	GA	High
Sharma 2015 [83]	Laparoscopic cholecystectomy	20/20	600 mg (600 mg)	Continuous	NSAID	GA	High
Srivastava 2009 [85]	Open cholecystectomy	60/60	600 mg	Single	Tramadol	GA	Low
Syal 2010 [86]	Open cholecystectomy	30/30	1200 mg	Single	Tramadol	GA	High
Takmaz 2009 [87]	Open cholecystectomy	30/15	900/1200 mg	Single	Tramadol Meripedine	GA	High
Hysterectomy trials							
Ajori 2011 [20]	Abdominal hysterectomy	69/69	600 mg	Single	Meripedine	GA	High
Badawy 2014 [23]	Hysterectomy	20/20	800 mg	Single	Meripedine Acetaminophen	GA	Unclear
Behdad 2012 [25]	Hysterectomy	30/31	100 mg (300 mg/d)	Continuous	An opioid	GA	High
Dierking 2003 [33]	Abdominal hysterectomy (salphinooophrectomy)	40/40	1200 mg (1800 mg/d)	Continuous	Morphine	GA	High
Durmus 2006 [36]	Hysterectomy	25/25	1200 mg	Single	Morphine	GA	Unclear
Fassoulaki 2005 [39]	Abdominal hysterectomy	29/30	400 mg (1600 mg/d)	Continuous	Morphine Paracetamol Codeine	GA	Low
Fassoulaki 2006 [40]	Abdominal hysterectomy	30/30	400 mg (1600 mg/d)	Continuous	Morphine Paracetamol Codeine Local anesthetic	GA	High
Frouzanfard 2013 [41]	Abdominal hysterectomy	25/25	1200 mg	Single	Morphine NSAID	GA	High
Ghafari 2009 [42]	Abdominal hysterectomy and salphinooophrectomy	33/33	300 mg (300 mg/d)	Continuous	Morphine	GA	Unclear
Ghai 2011 [43]	Abdominal hysterectomy	30/30	900 mg	Single	Morphine NSAID	GA	Low
Gilron 2004 [44]	Abdominal hysterectomy	23/24	1800 mg (1800 mg/d)	Continuous	Morphine	GA	High
Joseph 2014 [50]	Abdominal hysterectomy	25/25	600 mg	Single	Morphine	GA	High
Khan 2013 [53]	Abdominal hysterectomy	34/35	1200 mg	Single	Nalbuphine	GA	High
Ram 2015 [75]	Abdominal hysterectomy	30/30	900 mg	Single	NSAID	Spinal anesthesia	High
Ray 2015 [77]	Abdominal hysterectomy	30/30	300 mg	Single	NSAID	Spinal anesthesia	High
Rorarius 2004 [78]	Vaginal hysterectomy	45/45	600 mg	Single	Fentanyl	GA	High
Sekhavet 2009 [80]	Abdominal hysterectomy	49/49	600 mg (300 mg/d)	Continuous	Morphine NSAID	GA	Unclear
Sen 2009a [82]	Abdominal hysterectomy and salphinooophrectomy	20/20	1200 mg	Single	Morphine Acetaminophen Codeine	GA	High
Turan 2003a [88]	Abdominal hysterectomy and salphinooophrectomy	25/25	1200 mg	Single	Tramadol	GA	High
Turan 2006 [90]	Abdominal hysterectomy and salphinooophrectomy	25/25	1200 mg (1200 mg/d)	Continuous	Acetaminophen Codeine	GA	High
Verma 2008 [94]	Abdominal hysterectomy	25/25	300 mg	Single	Epidural analgesia	Spinal-epidural anesthesia	High
Mastectomy trials							
Amr 2009 [21]	Radical or partial mastectomy	50/50	300 mg (300 mg/d)	Continuous	Morphine Acetaminophen Codeine	GA	Unclear
Azemati 2013 [22]	Radical mastectomy or quandrandectomy and axillary node dissection	50/50	600 mg	Single	Pethidine Acetaminophen	GA	High
Bharti 2012 [28]	Total mastectomy with axillary node dissection	20/20	600 mg	Single	Morphine NSAID	GA	Unclear
Butt 2010 [29]	Mastectomy	50/50	1200 mg	Single	Morphine	GA	Unclear
Dirks 2002 [34]	Unilateral radical mastectomy with axillary dissection	31/34	1200 mg	Single	Morphine	GA	High
Doha 2010 [35]	Radical mastectomy	30/30	1200 mg	Single	Tramadol NSAID	GA	High
Fassoulaki 2002 [38]	Radical mastectomy or lobectomy with axillary lymph node dissection	25/25	400 mg (1200 mg/d)	Continuous	Propoxyphene Acetaminophen Codeine	GA	High
Gosai 2015 [45]	Radical mastectomy	30/30	600 mg	Single	NSAID	GA	High
Grover 2009 [47]	Total mastectomy with axillary node dissection	27/23	600 mg	Single	Morphine	GA	High
Kim 2004 [55]	Mastectomy	21/20	900 mg	Single	Fentanyl	GA	Unclear
Metry 2008 [62]	Unilateral radical mastectomy and axillary dissection	67/34	1200 mg	Single	Morphine	GA	High
Orthopedic arthroplasty surgery trials							
Clarke 2009a [30]	Total knee arthroplasty	29/7	600 mg (300/600/900 mg/d)	Single/continuous	Morphine NSAID Regional anesthesia	Spinal anesthesia and sedation	High
Clarke 2009b [31]	Total hip arthroplasty	76/39	600 mg	Single (pre/post-operative administration)	Morphine NSAID Acetaminophen Dexamethasone	Spinal anesthesia	Unclear
Clarke 2014 [32]	Total knee arthroplasty	95/84	600 mg	Single	Morphine NSAID Regional anesthesia	Spinal anesthesia and sedation	High
Lunn 2015 [59]	Total knee arthroplasty	186/99	900/600 mg (1300/900 mg/d)	Continuous	Sufentanil Oxycodone NSAID Acetaminophen Local infiltration analgesia	Spinal anesthesia and sedation	Low
Paul 2013 [73]	Total knee arthroplasty	52/49	600 mg (600 mg)	Continuous	Morphine NSAID Acetaminophen	Spinal anesthesia and sedation	Low
Paul 2015 [72]	Total hip arthroplasty	48/54	600 mg (600 mg)	Continuous	Morphine NSAID Acetaminophen	Spinal anesthesia and sedation	Low
Spinal surgery trials							
Erten 2010 [37]	Laminectomy	39/20	900/1200 mg	Single	Tramadol Pethidine NSAID	GA	High
Khan 2010 [52]	Laminectomy	150/25	600/900/1200 mg (600/900/1200 mg)	Continuous	Morphine	GA	Unclear
Khurana 2013 [54]	Discoidectomy	30/30	300 mg (900 mg/d)	Continuous	Tramadol NSAID	GA	Unclear
Leung 2006 [58]	Spine surgery	9/12	900 mg (900 mg/d)	Continuous	Hydromorphine	GA	Unclear
Özgencil 2011 [66]	Laminectomy or discoidectomy	30/30	1800 mg (1200 mg/d)	Continuous	Morphine	GA	Unclear
Panday 2004a [67]	Discoidectomy	80/20	300/600/900/1200 mg	Single	Fentanyl	GA	High
Panday 2004c [69]	Discoidectomy	28/28	300 mg	Single	Fentanyl	GA	Unclear
Radhakrishnan 2005 [74]	Laminectomy or Discoidectomy	30/30	800 mg (800 mg/d)	Continuous	Morphine	GA	High
Turan 2003b [89]	Discoidectomy or spinal fusion	25/25	1200 mg	Single	Morphine	GA	High
Vahedi 2011 [92]	Laminectomy or Discoidectomy	36/40	300 mg	Single	Morphine	GA	High
Vasigh 2015 [93]	Laminectomy	38/38	600 mg (900 mg/d)	Continuous	Morphine	GA	High
Thoracic surgery trials							
Grosen 2014 [46]	Thoracotomy for malignancies	52/52	1200 mg (increasing dose to 1200 mg/d)	Continuous	Morphine NSAID Acetaminophen Epidural analgesia	GA	Low
Hout 2007 [49]	Exploratory thoracotomy, pneumonectomy, lobectomy, segmentectomy, biopsy	23/28	1200 mg	Single	Hydromorphine Epidural analgesia	GA	High
Kinney 2011 [56]	Thoratectomy; Lobectomy; Wedge resection; Segmentectomy; Pneumonectomy; Chest wall resection	57/68	600 mg	Single	Fentanyl NSAID Acetaminophen Epidural analgesia	GA	Low
Kosucu 2013 [57]	Posterolateral or lateral thoracotomy	29/31	1200 mg	Single	Morphine Meriphidine NSAID	GA	High
Menda 2010 [61]	Coronary artery bypass graft	30/30	600 mg	Single	Morphine Acetaminophen	GA	Unclear
Omran 2005 [65]	Posterolateral thoracotomy for lobectomy	25/25	1200 mg (1200 mg/d)	Continuous	Morphine	GA	Unclear
Rapchuk 2009 [76]	Cardiac surgery via Sternum	27/27	1200 mg (600 mg/d)	Continuous	Fentanyl Acetaminophen	GA	High
Soltanzadeh 2011 [84]	Coronary artery bypass graft	30/30	800 mg (400 mg/d)	Continuous	Morphine	GA	High
Ucak 2011 [91]	Coronary artery bypass graft	20/20	1200 mg (1200 mg/d)	Continuous	Tramadol Acetaminophen	GA	High

^a^The continuous treatment is defined as more than one administration of gabapentin. The mg/day is the dose of gabapentin per day in the treatments that extends one administration

Sixty-six trials were classified as small trials [21–27, 29–31, 33–45, 47–55, 57, 58, 61–64, 66, 67, 69, 71–81, 83, 84, 86–94], five had more than 50 participants in each group [20, 32, 46, 56, 85], and three included more than 200 patients [59, 68, 70].

The gabapentin dose in the included trials ranged from 100 mg to 1800 mg, and was mostly administered as a single dose (46 trials). [20, 22–24, 28–32, 34–37, 41, 43, 45, 47–51, 53, 55–57, 61–64, 67–71, 75, 77–79, 81, 82, 85–90, 92, 94] In 30 trials, gabapentin was administered in combination with a basic, non-opioid/opioid analgesic regimen [20, 22, 23, 26, 28, 30–32, 37–41, 43, 46, 49, 54, 56, 57, 59, 61, 72, 73, 76, 79, 80, 82, 87, 90, 91]. In 44 trials, gabapentin was administered together with an opioid as the only analgesic [20, 25, 27, 29, 33–36, 44, 45, 47, 48, 50–53, 55, 58, 62–71, 74, 75, 77, 78, 83–86, 88, 89, 92–94]. In five trials, gabapentin was administered in combination with a NSAID [27, 45, 75, 77, 83],, and in two trials, the postoperative analgesic regimen was not described [24, 81].

### Bias assessments in surgical subgroups

Eight trials were classified as overall low risk of bias. None from the mastectomy subgroup and one trial from the cholecystectomy group was overall low risk of bias [85]. In the subgroups hysterectomy, [39, 43] and thoracic surgery [46, 56] two trials were low risk of bias in each group, and three trials were classified as low risk of bias in the orthopedic arthroplasty subgroup [59, 72, 73].

Below, we present analyses from trials with low risk of bias. In addition, analyses from all trials are presented in Table 2: Primary outcomes from trials with low risk of bias and all trials estimates, and Table 3: Secondary outcomes from trials with low risk of bias and all trials estimate.

**Table 2:** Primary outcomes from trials with low risk of bias and all trials estimates

Surgical procedure	Cholecystectomy		Hysterectomy		Mastectomy		Orthopedic arthroplasty surgery		Spinal Surgery		Thoracic surgery	
Outcomes	Reduction (mg)/RR *Estimate (95% CI; p-value; trials)*	Test for subgroup differences *P-value*	Reduction (mg) /RR *Estimate (95% CI; p-value; trials)*	Test for subgroup differences *P-value*	Reduction (mg) /RR *Estimate (95% CI; p-value; trials)*	Test for subgroup differences *P-value*	Reduction (mg) /RR *Estimate (95% CI; p-value; trials)*	Test for subgroup differences *P-value*	Reduction (mg) /RR *Estimate (95% CI; p-value; trials)*	Test for subgroup differences *P-value*	Reduction (mg)/RR *Estimate (95% CI; p-value; trials)*	Test for subgroup differences *P-value*
Beneficial outcomes												
24-h morphine consumption *Low risk of bias*	12.2 mg (9.8, 14.6; 1 trial)	-	1.6 mg (−4.8, 8.0; 2 trials)	*P* = 0.21	-	-	4.0 mg (−0.8, 8.7; 3 trials)	*P* = 0.75	-	-	6.7 mg (−2.0, 15.4; 1 trial)	-
24-h morphine consumption *All trials*	7.3 mg (95% CI: 4.6, 9.9; *p* < 0.00001; 10 trials; TSA adj.CI: 3.5, 11.0; 62.4%)	*P* = 0.9	10.5 mg (95% CI: 6.7, 14.4; *p* < 0.00001; 14 trials; TSA adj. CI: 3.0, 18.1; 30.2%)	*P* = 0.16	5.2 mg (95% CI: 0.9, 9.5; *p* = 0.02; 6 trials; TSA adj. CI: −4.6, 15.0; 24.3%)	*P* = 0.15	6.1 mg (95% CI: 0.2, 12.1; *p* = 0.04; 6 trials; TSA adj.CI: −18.1, 30.3; 5.8%)	*P* = 0.47	10.6 mg (95% CI: 2.1, 19.0; *p* = 0.01; 8 trials; TSA adj.CI: −24.1, 45.2; 6.1%)	*P* = 0.52	6.3 mg (95% CI: 2.9, 9.8; *p* = 0.0003; 7 trials; TSA adj.CI: −0.3, 12.3; 28.9%)	*P* = 0.25
Harmful outcomes												
Serious adverse events *Low risk of bias*	Not estimable	-	-	-	-	-	2.98 (0.36, 24.41; two trials)	*P* = 0.49	-	-	1.35 (0.69, 2.63; two trials)	*P* = 0.49
Serious adverse events *All trials*	Not estimable	-	0.55 (95% CI: 0.05, 5.61; p 0.61; 5 trials; TSA adj. CI -; 3.3%)	*P* = 0.16	Not estimable	-	2.98 (95% CI: 0.36, 24.41; *p* = 0.31; 2 trials; TSA adj. CI: -; 2.1%)	*P* = 0.3	Not estimable	-	1.06 (95% CI: 0.57, 1.74; *p* = 0.81; 4 trials; TSA adj.CI: 0.5, 2.1; 55.9%)	*P* = 0.72

**Table 3:** Secondary outcomes from trials with low risk of bias and all trials

Surgical procedure	Cholecystectomy		Hysterectomy		Mastectomy		Orthopedic arthroplasty surgery		Spinal Surgery		Thoracic surgery	
Outcomes	Reduction (mm)/RR *Estimate (95% CI; p-value; trials)*	Test for subgroup differences *P-value*	Reduction (mm)/RR *Estimate (95% CI; p-value; trials)*	Test for subgroup differences *P-value*	Reduction (mm)/RR *Estimate (95% CI; p-value; trials)*	Test for subgroup differences *P-value*	Reduction (mm)/RR *Estimate (95% CI; p-value; trials)*	Test for subgroup differences *P-value*	Reduction (mm)/RR *Estimate (95% CI; p-value; trials)*	Test for subgroup differences *P-value*	Reduction (mm)/RR Estimate (95% CI; *p*-value; trials)	Test for subgroup differences *P*-value
Beneficial outcomes												
6-h VAS at rest *Low risk of bias*	26.0 mm (24.8, 27.2; 1 trial)	-	3.0 mm (−12.9, 18.9; 1 trial)	-	-	-	-	-	-	-	5.0 mm (−0.7, 10.7; 2 trials)	*P* = 0.36
6-h VAS at rest *All trials*	13.6 mm (95% CI: −6.2, 33.4; *p* = 0.18; 3 trials; TSA adj. CI: -; 4.4%)	*P* = 0.87	16.4 mm (95% CI: 11.9, 21.0; *p* < 0.00001; 16 trials; TSA adj. CI: 11.9, 21.0; 104.3%)	*P* = 0.05	7.8 mm (95% CI: 1.9, 13.7; *p* = 0.01; 7 trials; TSA adj. CI: −1.1, 16.7; 51.9%)	*P* = 0.15	Not estimable	-	10.2 mm (95% CI: 2.3, 18.0; *p* = 0.01; 7 trials; TSA adj. CI: −5.7, 26.0; 29.6%)	*P* = 0.59	6.6 mm (95% CI: 0.8, 12.4; *p* = 0.05; 6 trials; TSA adj.CI: −3.0, 12.3; 59.2%)	*P* = 0.02
6-h VAS at mobilization *Low risk of bias*	11.5 mm (10.3, 12.7; 1 trial)	-	13.0 mm (−26.0, 52.0; 1 trial)	-	-	-	-	-	-	-	8.9 mm (1.6, 16.1; 1 trial)	-
6-h VAS at mobilization *All trials*	11.5 mm (95%CI: 10.3, 12.7; *p* < 0.00001; 1 trial; TSA adj. CI: -)	*P* = 0.04	8.5 mm (95%CI: 2.8, 14.2; *p* = 0.004; 6 trials; TSA adj.CI: 0.2, 16.7; 56.1%)	*P* = 0.97	5.2 mm (95%CI: −1.6, 12.0; *p* = 0.14; 6 trials; TSA adj.CI: −5.1, 15.7; 44.8%)	*P* = 0.07	Not estimable	-	10.0 mm (95%CI: 1.1, 19.0; *p* = 0.03; 1 trial; TSA adj. CI: -)	*P* = 0.73	10.9 mm (95%CI: 4.4, 17.4; *p* = 0.001; 4 trials; TSA adj.CI: 0.1, 21.7; 42.8%)	*P* = 0.41
24-h VAS at rest *Low risk of bias*	7.0 mm (5.8, 8.2; 1 trial)	-	9.0 mm (−2.8, 20.8; 1 trial)	-	-	-	0.9 mm (−6.3, 8.1; 1 trial)	-	-	-	4.2 mm (−0.2, 8.5; 2 trials)	*P* = 0.06
24-h VAS at rest *All trials*	3.2 mm (95% CI: −1.9, 8.4; *p* = 0.22; 3 trials; TSA adj.CI: −3.3, 9.8; 69.1%)	*P* = 0.1	10.5 mm (95% CI: 6.9, 14.2; *p* < 0.00001; 15 trials; TSA adj.CI: 6.7, 14.4; 136.3%)	*P* = 0.02	2.6 mm (95% CI: −3.2, 8.5; *p* = 0.2; 3 trials; TSA adj. CI: −3.2, 8.5; 112.9%)	*P* = 0.02	1.0 mm (95% CI: −1.7, 3.7; *p* = 0.45; 3 trials; TSA adj.CI: −3.9, 6.0; 248.0%)	*P* = 0.001	6.2 mm (95% CI: 0.9, 11.6; *p* = 0.02; 6 trials; TSA adj.CI: 0.9, 13.3; 64.1%)	*P* = 0.61	9.7 mm (95% CI: 2.4, 17.1; *p* = 0.01; 8 trials; TSA adj.CI: −4.2, 23.7; 33.6%)	*P* = 0.51
24-h VAS at mobilization *Low risk of bias*	16.0 mm (15.1, 16.9; 1 trial)	-	0.5 mm (−36.5, 37.5; 1 trial)	-	-	-	0.5 mm (−11.4, 12.3; 1 trial)	-	-	-	0.1 mm (−8.9, 9.1; 1 trial)	-
24-h VAS at mobilization *All trials*	16.0 mm (95% CI: 15.1, 16.9; *p* < 0.0001; 1 trial; TSA adj. CI: -)	*P* < 0.00001	7.7 mm (95% CI: −8.2, 23.6; *p* = 0.34; 5 trials; TSA adj.CI: −16.3, 25.4; 22.5%)	*P* = 0.91	3.1 mm (95% CI: −13.6, 19.8; *p* = 0.72; 3 trials; −65.1, 71.3; 6.5%)	*P* = 0.87	Increase 6.5 mm (95% CI: 3.0, 9.9; *p* = 0.0002; 2 trials; TSA adj.CI: +2.5, +10.5; 150.7%)	*P* = 0.0008	Not estimable	-	3.0 mm (95% CI: −4.5, 10.4; *p* = 0.43; 6 trials; TSA adj.CI: −11.2, 17.1; 33.1%)	*P* = 0.86
Harmful outcomes												
Nausea *Low risk of bias*	-	-	-	-	-	-	0.8 (0.6,1.0; 2 trials)	*P* = 0.5	-	-	0.7 (0.5, 0.9; 1 trial)	-
Nausea *All trials*	0.5 (95% CI: 0.25, 0.99; *p* = 0.05; 1 trial; TSA adj. CI: -)	*P* = 0.16	0.77 (95% CI: 0.63, 0.95; *p* = 0.01; 11 trials; TSA adj. CI: 0.6, 1.1; 49.4%)	*P* = 0.73	1.38 (95% CI: 0.85, 2.23; *p* = 0.19; 3 trials; TSA adj. CI: -; 2.69%)	*P* = 0.33	0.83 (95% CI: 0.66, 1.03; *p* = 0.08; 4 trials; TSA adj. CI: -; 79.7%)	*P* = 0.91	1.07 (95% CI: 0.68, 1.68; *p* = 0.78; 8 trials; TSA adj. CI: 0.4, 3.2; 22.8%)	*P* = 0.29	0.66 (95% CI: 0.5, 0.88; *p* = 0.005; 5 trials; TSA adj. CI: 0.4, 1.0; 49.9%)	*P* = 0.1
Vomiting *Low risk of bias*	-	-	-	-	-	-	-	-	-	-	1.1 (0.7, 1.6; 1 trial)	-
Vomiting *All trials*	0.5 (95%CI: 0.21, 1.16; *p* = 0.11; 1 trial; TSA adj. CI: -)	*P* = 0.28	0.71 (95% CI: 0.57, 0.9; *p* = 0.005; 9 trials; TSA adj. CI: 0.5, 1.0; 71.7%)	*P* = 0.61	0.81 (95% CI: 0.48, 1.37; *p* = 0.44; 5 trials; TSA adj. CI: 0.1, 6.9; 16.5%)	*P* = 0.92	0.64 (95% CI: 0.26, 1.59; *p* = 0.34; 1 trial; TSA adj. CI: -)	*P* = 0.66	0.61 (95% CI: 0.33, 1.12; *p* = 0.11; 7 trials; TSA adj. CI: 0.2, 2.3; 18.6%)	*P* = 0.51	1.01 (95% CI: 0.69, 1.49; *p* = 0.96; 5 trials; TSA adj. CI: 0.2, 4.9; 8.0%)	*P* = 0.32
Sedation *Low risk of bias*	1.8 (0.8, 3.9; 1 trial)	-	0.8 (0.4, 1.6; 1 trial)	-	-	-	0.9 (0.7, 1.2; 1 trial)	-	-	-	1.2 (0.7, 2.3; 2 trials)	*P* = 0.55
Sedation *All trials*	3.28 (95% CI: 1.55, 6.94; *p* = 0.002; 5 trials; TSA adj. CI: -; 3.9%)	*P* = 0.009	1.08 (95% CI: 0.81, 1.45; *p* = 0.61; 7 trials; TSA adj. CI: 0.5, 2.2; 23.2%)	*P* = 0.06	1.04 (95% CI: 0.75, 1.44; *p* = 0.83; 2 trials; TSA adj. CI: -; 4.8%)	*P* = 0.06	0.97 (95% CI: 0.76, 1.24; *p* = 0.82; 2 trials; TSA adj. CI: 0.7, 1.6; 44.3%)	*P* = 0.01	2.65 (95% CI: 0.94, 7.52; *p* = 0.07; 7 trials; TSA adj. CI: -; 2.3%)	*P* = 0.19	1.34 (95% CI: 0.78, 2.32; *p* = 0.29; 5 trials; TSA adj. CI: 0.1, 12.5; 10.8%)	*P* = 0.68
Dizziness *Low risk of bias*	1.0 (0.7, 1.4; 1 trial)	-	6.2 (1.1, 34.0; 1 trial)	-	-	-	0.7 (0.2, 2.1; 1 trial)	-	-	-	1.0 (0.8, 1.3; 2 trials)	*P* = 0.64
Dizziness *All trials*	0.7 (95% CI: 0.52, 0.94; *p* = 0.02; 6 trials; TSA adj. CI: 0.2, 2.6; 27.1%)	*P* = 0.01	1.34 (95% CI: 0.95, 1.89; *p* = 0.1; 11 trials; TSA adj. CI: 0.3, 5.6; 16.8%)	*P* = 0.2	1.03 (95% CI: 0.74, 1.43; *p* = 0.88; 5 trials; TSA adj. CI: 0.6, 2.1; 31.4%)	*P* = 0.84	0.72 (95% CI: 0.32, 1.66; *p* = 0.45; 3 trials; TSA adj. CI: 0.0, 18.1; 5.6%)	*P* = 0.44	1.49 (95% CI: 0.77, 2.86; *p* = 0.24; 6 trials; TSA adj. CI: 0.6, 3.1; 31.4%)	*P* = 0.22	1.04 (95% CI: 0.85, 1.26; *p* = 0.7; 4 trials; TSA adj. CI: 0.7, 1.5; 36.4%)	*P* = 0.66

### Primary outcomes

#### 24-h morphine consumption

24-h morphine consumption was reported in 51 trials with 4193 patients. [21, 23, 26, 28, 30–33, 35–44, 46, 48–53, 55, 57, 59–63, 65–70, 72, 73, 80, 82, 84–89, 91–93] Of these 51 trials, 7 were classified as overall low risk of bias [39, 43, 46, 59, 72, 73, 85].

**Fig. 4:**
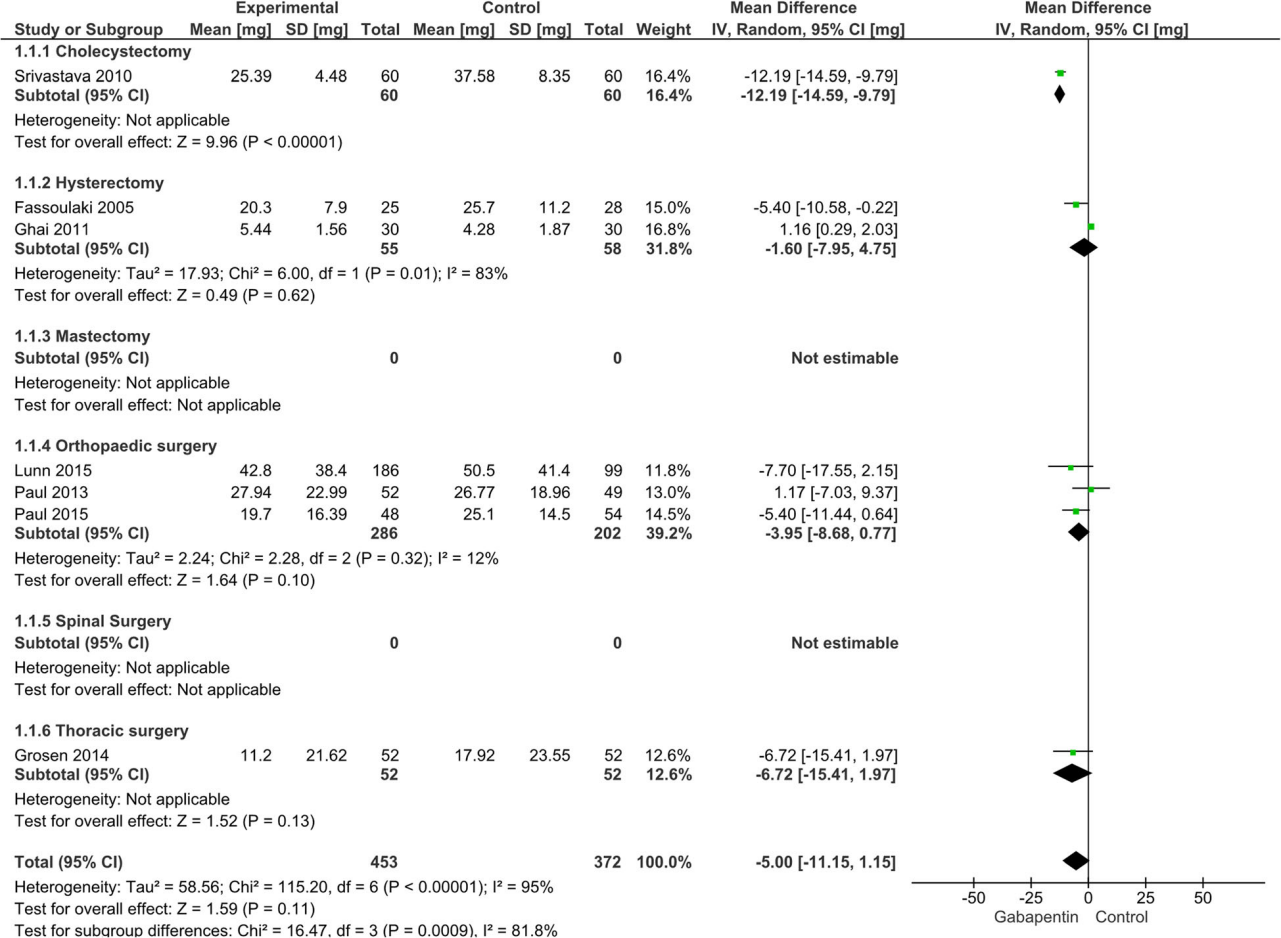
Forest plot of 24-h morphine consumption. Forest plot of 24-h morphine consumption of trials with overall low risk of bias

In cholecystectomy, one trial reported a reduction of 12.2 mg [9.8, 14.6] in 24-h morphine consumption in the gabapentin treatment group compared to controls [85], two trials in hysterectomy found a reduction of 1.6 mg [−4.8, 8.0] [39, 43], and three trials in orthopedic arthroplasty demonstrated a reduction of 4.0 mg [−0.8, 8.7] [58, 71, 72]. Finally, one trial in thoracic surgery reported a reduction of 6.7 mg [−2.0, 15.4] [46]. We found no difference between the surgical procedures when tested for subgroup differences.

(Table 2: The intervention effect estimated from trials with low risk of bias, and from all trials despite risk of bias; Fig. 4: Forest plot of 24-h morphine consumption from trials with low risk of bias).

(Additional file 1: Appendix 3 - Forest plot of 24-h morphine consumption from all trials, Additional file 1: Appendix 4–9 - TSA of estimates from all trials and cholecystectomy, hysterectomy, mastectomy, orthopedic arthroplasty, spinal and thoracic surgery groups).

#### Serious adverse events

Fifteen trials with 1377 patients reported SAEs [20, 33, 34, 38, 39, 44, 46, 49, 53, 56, 59, 72, 85, 91, 92]. Of the 15 trials, 5 were classified as overall low risk of bias [46, 56, 59, 72, 85]. The reported SAEs were: Death, urticarial rash, re-operation, prolonged admission, re-admission to hospital, pneumonia, and atrial fibrillation.

**Fig. 5:**
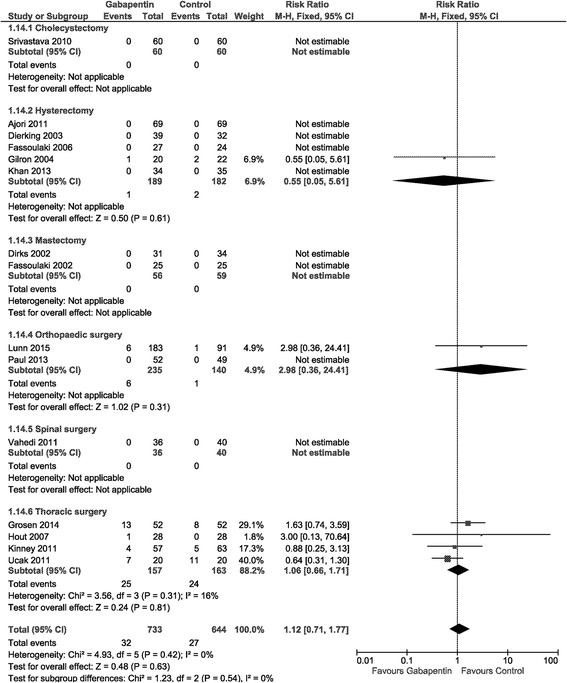
Forest plot of serious adverse events. Forest plot of Serious Adverse Events of trials with overall low risk of bias

One cholecystectomy trial, two orthopedic arthroplasty trials, and two thoracic surgery trials were classified as overall low risk of bias. [46, 56, 59, 72, 85] In the trials with low risk of bias, the risk of SAE’s were 2.98 [0.36, 24.41] in the orthopedic arthroplasty subgroup [59, 72] and 1.35 [0.69, 2.63] in the thoracic subgroup [46, 56]. A comparison of pooled-estimates in test for subgroup differences from trials with low risk of bias indicated no difference between groups, *p* =  0.49.

(Table 2: SAEs estimated from trials with low risk of bias, and from all trials despite risk of bias; Fig. 5: Forest plot of SAEs from trials with low risk of bias.

(Additional file 1: Appendix 10 - Forest plot of SAEs in all trials, Appendix 11 - TSA of SAEs in the thoracic surgery subgroup).

### Secondary outcomes

#### Pain intensity

In general, only few data were available from trials with low risk of bias, rendering tests for subgroup differences impossible and/or unreliable.

(Table 3: The intervention effect estimated from trials with low risk of bias, and from all trials despite risk of bias).

(Additional file 1: Appendix 12–15 - Forest plots of VAS 6 h postoperative at rest and mobilization, 24 h postoperative at rest and mobilization, all trials estimates).

#### Adverse events

No subgroup differences were demonstrated in any adverse event in trials with low risk of bias.

(Table 3: Adverse events estimated from trials with low risk of bias, and from all trials despite risk of bias).

(Additional file 1: Appendix 16–19 - Forest plots of nausea, vomiting, sedation and dizziness, all trials).

## Discussion

It has been argued that postoperative pain treatment should be “procedure-specific”, since different analgesics may have specific effects dependent on the surgical procedure [4, 5]. In the present, preplanned subgroup analyses, we aimed to compare the effects of perioperative gabapentin on postoperative opioid consumption, pain intensity, and adverse- and serious adverse events in six different surgical procedures. Our primary outcomes were 24-h morphine consumption and the risk of SAEs.

Our results are limited by the fact that overall, only eight trials were classified as overall low risk of bias, limiting our ability to test for subgroup differences, and to pool estimates in meta-analyses of these eight trials. When interpreting the results from the all trials analyses, it should be noted that about two-thirds of these trials had overall high risk of bias, which is a severe limitation to any conclusion on the outcomes.

In trials with low risk of bias, 24-h morphine consumption varied, and only the cholecystectomy subgroup indicated a difference between groups. With only one trial in this subgroup, the result has not been reproduced, and is difficult to interpret.

For the analysis of all trials, the difference in 24-h morphine consumption between surgical procedures was not statistically significant, when tested for subgroup differences. A reduction in 24-h morphine consumption was demonstrated for all surgical procedures compared with controls. However, the TSA did not reach required information size in any subgroup. Consequently, the effects observed in the individual procedures may be due to both random and systematic error, as indicated in the main systematic review [12].

SAEs were primarily reported in the thoracic surgery trials but overall, since SAEs were very poorly reported and data was sparse, it is not possible to conclude on this outcome.

For pain intensity outcomes, very few data were available from trials with low risk of bias. In the analyses of data from all trials, the results were divergent across surgical subgroups, and it is difficult to interpret the direction and authenticity in the test for subgroup differences.

No subgroup difference was demonstrated for any adverse event in trials with low risk of bias, and results from data including all trials were divergent across surgical subgroups, with no consistent differences in adverse events between surgical procedures. This indicates a similar adverse event profile of gabapentin for postoperative pain management irrespective of surgical procedure. Much like the previous outcomes, there is far too few data to firmly conclude based on these results. Poor reporting and high risk of bias limits any interpretation.

### Strengths and limitations of the subgroup analyses

These subgroup analyses have some strength. The analyses were planned in a PROSPERO published protocol, and were derived from a PRISMA compliant systematic review adhering to Cochrane standards in methodology and bias assessment. The trials have been critically assessed using the Cochrane bias assessment tools, and conclusions are based on trials with low risk of bias, which is unlike most of the previous systematic reviews. The TSA has been added to adjust for sparse data and repetitive resting, which is a risk when the vast majority of included trials are small, that is <50 patients in each group [95–98].

The limitations of this analysis mirror those of the included trials, and the limitations of the general methodology in subgroup analyses. Subgroup comparisons are to be perceived as observational because we compare pre-existing non-randomized groups, and must be interpreted as such [14].

The critical assessment of the trial methodology shows a very small number of trials with overall low risk of bias. Eighty-nine percent of the included trials have unclear or high risk of bias in one of the bias domains or more, risking an overestimation of beneficial -, and underestimation of harmful outcomes.

Despite the larger number of included trials in each subgroup compared with previous published systematic reviews, there is still a risk of spurious results due to lack of sufficient data. The lack of statistically significant *p*-values in the present subgroup analyses may be due to a small effect size, or poor power to detect a large effect.

According to Oxman and Guyatt [99], Xin Sun et al [100] and their criteria to evaluate the credibility of subgroup analyses, we have to consider further limitations such as: If the subgroup can be considered independent; no a-priori direction of the subgroup effect has been published; the subgroup effects found in our analyses does not seem to consequently manifest in closely related outcomes.

### Relation to the previously published systematic reviews

A number of systematic reviews investigating individual surgical procedures, or with a procedure specific approach, have been published [95–98, 101–103]. Overall, there are some general methodological differences that separates the present work from previously published systematic reviews, such as: The emphasis on trials with low risk of bias, subgroup analyses with test for subgroup differences, and the use of trial sequential analyses. The emphasis on trials with low risk of bias prevents any direct comparison with estimates from other systematic reviews. Several reviews have evaluated bias in the included trials, and report different overall bias evaluations and number of trials with overall low risk of bias, compared to the present review [97, 98, 104]. This may be explained by the fact that that we contacted every author whenever a bias domain was deemed unclear risk of bias, to give them a chance to describe their methods in greater detail. To our knowledge, this was not done in the previously published systematic reviews.

In a recent review, the effect of trials with high risk of bias were tested using sensitivity analyses, and no impact was demonstrated on the outcomes [104]. This is in contrast to the present analyses, and to the findings of our recently published systematic review [12, 104]. The different approach to author contact, and a different statistical approach, may explain some of the differences in the findings. Further, the systematic review by Doleman et al. [104] reports that the type of surgery was not independently associated with the effect of gabapentin, which may provide some confirmation of the results from the present analyses. However, the results are obtained with different statistical approaches and the number and types of surgeries are not stated in the findings from Doleman et al. Consequently, the confirmation must be interpreted with caution [104]. In another review, Mathiesen et al. demonstrated a greater beneficial effect (reduction in 24-h opioid consumption) in their hysterectomy and spinal surgery groups [95], which seems similar to the results from the all trials estimates in Table 2 in the present review. Mathiesen et al., however, did not test for subgroup differences [95], and we found no differences between subgroups in the all trials estimates of the present review.

Most previous systematic reviews report favorable results for gabapentin treatment, similar to the findings from the all trials estimates in the subgroup analyses of the present review. In comparison with the systematic reviews of gabapentin for hysterectomy, cholecystectomy and thoracic surgeries [95, 96, 101], more trials have been included in our subgroups. Due to different inclusion criteria and subgroup analyses in the different published systematic reviews, it is not possible to conduct a full comparison of estimates.

However, none of the systematic reviews above have investigated the risk of SAEs, limiting the ability to weigh the benefit and harm of gabapentin in perioperative pain management [95–98, 101].

### Impact of the analyses

We observed no systematic differences in postoperative opioid consumption, pain intensity, or adverse- or serious adverse events between six different surgical procedures treated with peri-operative gabapentin.

SAEs were very poorly reported, and only half the subgroups reported this outcome. More than 80% of the SAEs were reported in the thoracic surgery trials, making it impossible to rely on the risk and subgroup differences between the surgical procedures. In the original review, excess SAEs were reported in the gabapentin versus control groups, and approximately twice as many SAEs were found in trials with low risk of bias, compared with all trials [13]. Most trials have a short follow-up period and only report on SAEs and adverse effects for a short period postoperatively, which seems insufficient for a full evaluation. The inconsequent and diverse reporting of SAEs and adverse events complicates any reliable evaluation of these outcomes.

## Conclusion

Both beneficial and harmful effects in the present subgroup analyses are influenced by bias and insufficient data, limiting any conclusion. The very poorly reported incidence of SAEs limits any conclusion based on this outcome. Because of these limitations, we could not properly test for any major differences in beneficial or adverse outcomes between various surgical subgroups with gabapentin for postoperative pain. Consequently, our analyses cannot confirm or reject the concept of a procedure specific effect of gabapentin on postoperative pain.

## Additional files

Additional file 1: **Appendix 1.** Search strategies. **Appendix 2.** Opioid conversion table. **Appendix 3.** Forest plot of 24-h morphine consumption from all trials estimates. **Appendix 4.** TSA of 24-h morphine consumption in the cholecystectomy subgroup, all trials. **Appendix 5.** TSA of 24-h morphine consumption in the hysterectomy subgroup, all trials. **Appendix 6.** TSA of 24-h morphine consumption in the mastectomy subgroup, all trials. **Appendix 7.** TSA of 24-h morphine consumption in the orthopedic arthroplasty subgroup, all trials. **Appendix 8.** TSA of 24-h morphine consumption in the spinal subgroup, all trials. **Appendix 9.** TSA of 24-h morphine consumption in the thoracic surgery subgroup, all trials. **Appendix 10.** Forest plot of SAEs, all trials. **Appendix 11.** TSA of SAEs in the thoracic surgery subgroup, all trials. **Appendix 12.** Forest plot of VAS 6 h at rest, all trials. **Appendix 13.** Forest plot of VAS 6 h at mobilization, all trials. **Appendix 14.** Forest plot of VAS 24 h at rest, all trials. **Appendix 15.** Forest plot of VAS 24 h at mobilization, all trials. **Appendix 16.** Forest plot nausea, all trials. **Appendix 17.** Forest plot vomiting, all trials. **Appendix 18.** Forest plot sedation, all trials. **Appendix 19.** Forest plot dizziness, all trials. (PDF 736 kb)

## Abbreviations

CI: Confidence interval
D: Day
GA: General anesthesia
ICH-GCP: International committee of harmonization – good clinical practice
ICMJE: International committee of medical journal editors
NSAID: None-steroid anti-inflammatory drugs
PRISMA: Preferred reporting items for systematic reviews and meta-analyses
RCT: Randomized, clinical trial
SAE: Serious adverse events
TSA: Trial sequential analysis
