# Supplementary material for: Gabapentin in procedure-specific postoperative pain management – preplanned subgroup analyses from a systematic review with meta-analyses and trial sequential analyses
Source: BMC Anesthesiol. 2017 Jun 21;17:85. doi: 10.1186/s12871-017-0373-8 (PMC5480107; doi:10.1186/s12871-017-0373-8)

ADDITIONAL FILE 1

Appendix 1: Search strategies

Appendix 2: Opioid conversion table

Appendix 3: Forest plot of 24-hour morphine consumption from all trials estimates

Appendix 4: TSA of 24-hour morphine consumption in the cholecystectomy subgroup, all trials

Appendix 5: TSA of 24-hour morphine consumption in the hysterectomy subgroup, all trials

Appendix 6: TSA of 24-hour morphine consumption in the mastectomy subgroup, all trials

Appendix 7: TSA of 24-hour morphine consumption in the orthopedic arthroplasty subgroup, all trials

Appendix 8: TSA of 24-hour morphine consumption in the spinal subgroup, all trials

Appendix 9: TSA of 24-hour morphine consumption in the thoracic surgery subgroup, all trials

Appendix 10: Forest plot of SAEs, all trials

Appendix 11: TSA of SAEs in the thoracic surgery subgroup, all trials

Appendix 12: Forest plot of VAS 6 hours at rest, all trials

Appendix 13: Forest plot of VAS 6 hours at mobilization, all trials

Appendix 14: Forest plot of VAS 24 hours at rest, all trials

Appendix 15: Forest plot of VAS 24 hours at mobilization, all trials

Appendix 16: Forest plot nausea, all trials

Appendix 17: Forest plot vomiting, all trials

Appendix 18: Forest plot sedation, all trials

Appendix 19: Forest plot dizziness, all trials

Appendix 1: Search strategies

Preliminary searches performed 13 November 2013

Total number of references identified: 16895 references
Number of duplicates removed: 3189 references
Number of references in final list: 13706 references

Batch name: 131113_J Wetterslev_GABA

Cochrane Central Register of Controlled Trials (CENTRAL)(Issue 10 of 12, 2013) in The Cochrane Library (2411 hits in CENTRAL)
#1 MeSH descriptor: [Amines] explode all trees and with qualifiers: [Adverse effects - AE, Therapeutic use - TU]
#2 MeSH descriptor: [gamma-Aminobutyric Acid] explode all trees and with qualifiers: [Adverse effects - AE, Therapeutic use - TU]
#3 MeSH descriptor: [Cyclohexanecarboxylic Acids] explode all trees and with qualifiers: [Adverse effects - AE, Therapeutic use - TU]
#4 (gaba* or neurontin* or neurotonin* or horizant*)
#5 #1 or #2 or #3 or #4
#6 MeSH descriptor: [Pain] explode all trees
#7 pain*
#8 #6 or #7
#9 #5 and #8
#10 adult* or middle age* or aged
#11 #9 and #10

MEDLINE (Ovid SP)(1946 to November 2013)(7072 hits)
1. exp Amines/ae, tu [Adverse Effects, Therapeutic Use]
2. exp gamma-Aminobutyric Acid/ae, tu [Adverse Effects, Therapeutic Use]
3. exp Cyclohexanecarboxylic Acids/ae, tu [Adverse Effects, Therapeutic Use]
4. (gaba* or neurontin* or neurotonin* or horizant*).mp. [mp=title, abstract, original title, name of substance word, subject heading word, keyword heading word, protocol supplementary concept, rare disease supplementary concept, unique identifier]
5. 1 or 2 or 3 or 4
6. exp Pain/
7. pain*.mp. [mp=title, abstract, original title, name of substance word, subject heading word, keyword heading word, protocol supplementary concept, rare disease supplementary concept, unique identifier]
8. 6 or 7
9. 5 and 8
10. limit 9 to (humans and ("all adult (19 plus years)" or "young adult (19 to 24 years)" or "adult (19 to 44 years)" or "young adult and adult (19-24 and 19-44)" or "middle age (45 to 64 years)" or "middle aged (45 plus years)" or "all aged (65 and over)" or "aged (80 and over)"))

EMBASE (1974 to November 2013)(3653 hits)
1. amine/ae, dt, th [Adverse Drug Reaction, Drug Therapy, Therapy]
2. 4 aminobutyric acid/ae, dt [Adverse Drug Reaction, Drug Therapy]
3. cyclohexanecarboxylic acid derivative/ae, dt [Adverse Drug Reaction, Drug Therapy]
4. (gaba* or neurontin* or neurotonin* or horizant*).mp. [mp=title, abstract, subject headings, heading word, drug trade name, original title, device manufacturer, drug manufacturer, device trade name, keyword]
5. 1 or 2 or 3 or 4
6. exp pain/

7. pain*.mp. [mp=title, abstract, subject headings, heading word, drug trade name, original title, device manufacturer, drug manufacturer, device trade name, keyword]
8. 6 or 7
9. 5 and 8
10. limit 9 to (human and (adult <18 to 64 years> or aged <65+ years>))

Science Citation Index Expanded (http://apps.webofknowledge.com)(1900 to November 2013)(3759 hits)
#3 3,759 #2 AND #1
#2 385,187 TS=(pain*)
#1 68,630 TS=(gaba* or neurontin* or neurotonin* or horizant*)

Preliminary searches performed 30 June 2014

Total number of references identified: 16861 references
Number of duplicates removed: 3592 references
Number of references in final list: 13569 references
Number of new references: 789 references

Batch name: 140701_J Wetterslev_GABA

Cochrane Central Register of Controlled Trials (CENTRAL)(Issue 6 of 12, 2014) in The Cochrane Library (2619 hits in CENTRAL)
#1 MeSH descriptor: [Amines] explode all trees and with qualifiers: [Adverse effects - AE, Therapeutic use - TU]
#2 MeSH descriptor: [gamma-Aminobutyric Acid] explode all trees and with qualifiers: [Adverse effects - AE, Therapeutic use - TU]
#3 MeSH descriptor: [Cyclohexanecarboxylic Acids] explode all trees and with qualifiers: [Adverse effects - AE, Therapeutic use - TU]
#4 (gaba* or neurontin* or neurotonin* or horizant*)
#5 #1 or #2 or #3 or #4
#6 MeSH descriptor: [Pain] explode all trees
#7 pain*
#8 #6 or #7
#9 #5 and #8
#10 adult* or middle age* or aged
#11 #9 and #10

MEDLINE (Ovid SP)(1946 to July 2014)(6319 hits)
1. exp Amines/ae, tu [Adverse Effects, Therapeutic Use]
2. exp gamma-Aminobutyric Acid/ae, tu [Adverse Effects, Therapeutic Use]
3. exp Cyclohexanecarboxylic Acids/ae, tu [Adverse Effects, Therapeutic Use]
4. (gaba* or neurontin* or neurotonin* or horizant*).mp. [mp=title, abstract, original title, name of substance word, subject heading word, keyword heading word, protocol supplementary concept, rare disease supplementary concept, unique identifier]
5. 1 or 2 or 3 or 4
6. exp Pain/
7. pain*.mp. [mp=title, abstract, original title, name of substance word, subject heading word, keyword heading word, protocol supplementary concept, rare disease supplementary concept, unique identifier]
8. 6 or 7
9. 5 and 8
10. limit 9 to (humans and ("all adult (19 plus years)" or "young adult (19 to 24 years)" or "adult (19 to 44 years)" or "young adult and adult (19-24 and 19-44)" or "middle age (45 to 64 years)" or "middle aged (45 plus years)" or "all aged (65 and over)" or "aged (80 and over)"))

EMBASE (1974 to July 2014)(3847 hits)
1. amine/ae, dt, th [Adverse Drug Reaction, Drug Therapy, Therapy]
2. 4 aminobutyric acid/ae, dt [Adverse Drug Reaction, Drug Therapy]
3. cyclohexanecarboxylic acid derivative/ae, dt [Adverse Drug Reaction, Drug Therapy]
4. (gaba* or neurontin* or neurotonin* or horizant*).mp. [mp=title, abstract, subject headings, heading word, drug trade name, original title, device manufacturer, drug manufacturer, device trade name, keyword]
5. 1 or 2 or 3 or 4
6. exp pain/
7. pain*.mp. [mp=title, abstract, subject headings, heading word, drug trade name, original title, device manufacturer, drug manufacturer, device trade name, keyword]
8. 6 or 7
9. 5 and 8
10. limit 9 to (human and (adult <18 to 64 years> or aged <65+ years>))

Science Citation Index Expanded (http://apps.webofknowledge.com)(1900 to July 2014)(4076 hits)
#3 4,076 #2 AND #1
#2 417,945 TS=(pain*)
#1 72,059 TS=(gaba* or neurontin* or neurotonin* or horizant*)

Preliminary searches performed 14 November 2014

Total number of references identified: 17315 references
Number of duplicates removed: 4105 references
Number of references in final list: 13210 references
Number of new references: 462 references

Batch name: 141114_J Wetterslev_GABA NEW

Cochrane Central Register of Controlled Trials (CENTRAL)(Issue 11 of 12, 2014) (2645 hits)
#1 MeSH descriptor: [Amines] explode all trees and with qualifiers: [Adverse effects - AE, Therapeutic use - TU]
#2 MeSH descriptor: [gamma-Aminobutyric Acid] explode all trees and with qualifiers: [Adverse effects - AE, Therapeutic use - TU]
#3 MeSH descriptor: [Cyclohexanecarboxylic Acids] explode all trees and with qualifiers: [Adverse effects - AE, Therapeutic use - TU]
#4 (gaba* or neurontin* or neurotonin* or horizant*)
#5 #1 or #2 or #3 or #4
#6 MeSH descriptor: [Pain] explode all trees
#7 pain*
#8 #6 or #7
#9 #5 and #8
#10 adult* or middle age* or aged
#11 #9 and #10

MEDLINE (Ovid SP)(1946 to November 2014)(6549 hits)
1. exp Amines/ae, tu [Adverse Effects, Therapeutic Use]
2. exp gamma-Aminobutyric Acid/ae, tu [Adverse Effects, Therapeutic Use]
3. exp Cyclohexanecarboxylic Acids/ae, tu [Adverse Effects, Therapeutic Use]
4. (gaba* or neurontin* or neurotonin* or horizant*).mp. [mp=title, abstract, original title, name of substance word, subject heading word, keyword heading word, protocol supplementary concept, rare disease supplementary concept, unique identifier]
5. 1 or 2 or 3 or 4

6. exp Pain/
7. pain*.mp. [mp=title, abstract, original title, name of substance word, subject heading word, keyword heading word, protocol supplementary concept, rare disease supplementary concept, unique identifier]
8. 6 or 7
9. 5 and 8
10. limit 9 to (humans and ("all adult (19 plus years)" or "young adult (19 to 24 years)" or "adult (19 to 44 years)" or "young adult and adult (19-24 and 19-44)" or "middle age (45 to 64 years)" or "middle aged (45 plus years)" or "all aged (65 and over)" or "aged (80 and over)"))

<u>EMBASE (1974 to November 2014)(3962 hits)</u>
1. amine/ae, dt, th [Adverse Drug Reaction, Drug Therapy, Therapy]
2. 4 aminobutyric acid/ae, dt [Adverse Drug Reaction, Drug Therapy]
3. cyclohexanecarboxylic acid derivative/ae, dt [Adverse Drug Reaction, Drug Therapy]
4. (gaba* or neurontin* or neurotonin* or horizant*).mp. [mp=title, abstract, subject headings, heading word, drug trade name, original title, device manufacturer, drug manufacturer, device trade name, keyword]
5. 1 or 2 or 3 or 4
6. exp pain/
7. pain*.mp. [mp=title, abstract, subject headings, heading word, drug trade name, original title, device manufacturer, drug manufacturer, device trade name, keyword]
8. 6 or 7
9. 5 and 8
10. limit 9 to (human and (adult <18 to 64 years> or aged <65+ years>))

<u>Science Citation Index Expanded (1900 to November 2014)(4159 hits)</u>
#3 4,159 #2 AND #1
#2 417,588 TS=(pain*)
#1 72,305 TS=(gaba* or neurontin* or neurotonin* or horizant*)

Preliminary searches performed 9 April 2015

Total number of references identified: 17466 references
Number of duplicates removed: 4042 references
Number of references in final list: 13424 references
Number of new references: 126 references

Batch name: 150409_J Wetterslev_GABA

<u>Cochrane Central Register of Controlled Trials (CENTRAL) (Issue 3 of 12, 2015) (2629 hits)</u>
#1 MeSH descriptor: [Amines] explode all trees and with qualifiers: [Adverse effects - AE, Therapeutic use - TU]
#2 MeSH descriptor: [gamma-Aminobutyric Acid] explode all trees and with qualifiers: [Adverse effects - AE, Therapeutic use - TU]
#3 MeSH descriptor: [Cyclohexanecarboxylic Acids] explode all trees and with qualifiers: [Adverse effects - AE, Therapeutic use - TU]
#4 (gaba* or neurontin* or neurotonin* or horizant*)
#5 #1 or #2 or #3 or #4
#6 MeSH descriptor: [Pain] explode all trees
#7 pain*
#8 #6 or #7
#9 #5 and #8
#10 adult* or middle age* or aged
#11 #9 and #10

MEDLINE (Ovid SP)(1946 to April 2015) (6432 hits)
1. exp Amines/ae, tu [Adverse Effects, Therapeutic Use]
2. exp gamma-Aminobutyric Acid/ae, tu [Adverse Effects, Therapeutic Use]
3. exp Cyclohexanecarboxylic Acids/ae, tu [Adverse Effects, Therapeutic Use]
4. (gaba* or neurontin* or neurotonin* or horizant*).mp. [mp=title, abstract, original title, name of substance word, subject heading word, keyword heading word, protocol supplementary concept, rare disease supplementary concept, unique identifier]
5. 1 or 2 or 3 or 4
6. exp Pain/
7. pain*.mp. [mp=title, abstract, original title, name of substance word, subject heading word, keyword heading word, protocol supplementary concept, rare disease supplementary concept, unique identifier]
8. 6 or 7
9. 5 and 8
10. limit 9 to (humans and ("all adult (19 plus years)" or "young adult (19 to 24 years)" or "adult (19 to 44 years)" or "young adult and adult (19-24 and 19-44)" or "middle age (45 to 64 years)" or "middle aged (45 plus years)" or "all aged (65 and over)" or "aged (80 and over)"))

EMBASE (1974 to April 2015) (4081 hits)
1. amine/ae, dt, th [Adverse Drug Reaction, Drug Therapy, Therapy]
2. 4 aminobutyric acid/ae, dt [Adverse Drug Reaction, Drug Therapy]
3. cyclohexanecarboxylic acid derivative/ae, dt [Adverse Drug Reaction, Drug Therapy]
4. (gaba* or neurontin* or neurotonin* or horizant*).mp. [mp=title, abstract, subject headings, heading word, drug trade name, original title, device manufacturer, drug manufacturer, device trade name, keyword]
5. 1 or 2 or 3 or 4
6. exp pain/
7. pain*.mp. [mp=title, abstract, subject headings, heading word, drug trade name, original title, device manufacturer, drug manufacturer, device trade name, keyword]
8. 6 or 7
9. 5 and 8
10. limit 9 to (human and (adult <18 to 64 years> or aged <65+ years>))

Science Citation Index Expanded (1900 to April 2015) (4324 hits)
#3 4,324 #2 AND #1
#2 430,421 TS=(pain*)
#1 73,791 TS=(gaba* or neurontin* or neurotonin* or horizant*)

Preliminary searches performed 23rd September 2015
Total number of references identified: 18200 references
Number of duplicates removed: 4184 references
Number of references in final list: 14016 references
Number of new references: 1188 references

Batch name: 150915_J Wetterslev_GABA

Cochrane Central Register of Controlled Trials (CENTRAL) (Issue 8 of 12, 2015) (2798 hits)
#1 MeSH descriptor: [Amines] explode all trees and with qualifiers: [Adverse effects - AE, Therapeutic use - TU]
#2 MeSH descriptor: [gamma-Aminobutyric Acid] explode all trees and with qualifiers: [Adverse effects - AE, Therapeutic use - TU]
#3 MeSH descriptor: [Cyclohexanecarboxylic Acids] explode all trees and with qualifiers: [Adverse effects - AE, Therapeutic use - TU]
#4 (gaba* or neurontin* or neurotonin* or horizant*)
#5 #1 or #2 or #3 or #4

#6 MeSH descriptor: [Pain] explode all trees
#7 pain*
#8 #6 or #7
#9 #5 and #8
#10 adult* or middle age* or aged
#11 #9 and #10

MEDLINE (Ovid SP) (1946 to September 2015) (6621 hits)
1. exp Amines/ae, tu [Adverse Effects, Therapeutic Use]
2. exp gamma-Aminobutyric Acid/ae, tu [Adverse Effects, Therapeutic Use]
3. exp Cyclohexanecarboxylic Acids/ae, tu [Adverse Effects, Therapeutic Use]
4. (gaba* or neurontin* or neurotonin* or horizant*).mp. [mp=title, abstract, original title, name of substance word, subject heading word, keyword heading word, protocol supplementary concept, rare disease supplementary concept, unique identifier]
5. 1 or 2 or 3 or 4
6. exp Pain/
7. pain*.mp. [mp=title, abstract, original title, name of substance word, subject heading word, keyword heading word, protocol supplementary concept, rare disease supplementary concept, unique identifier]
8. 6 or 7
9. 5 and 8
10. limit 9 to (humans and ("all adult (19 plus years)" or "young adult (19 to 24 years)" or "adult (19 to 44 years)" or "young adult and adult (19-24 and 19-44)" or "middle age (45 to 64 years)" or "middle aged (45 plus years)" or "all aged (65 and over)" or "aged (80 and over)"))

EMBASE (1974 to September 2015) (4289 hits)
1. amine/ae, dt, th [Adverse Drug Reaction, Drug Therapy, Therapy]
2. 4 aminobutyric acid/ae, dt [Adverse Drug Reaction, Drug Therapy]
3. cyclohexanecarboxylic acid derivative/ae, dt [Adverse Drug Reaction, Drug Therapy]
4. (gaba* or neurontin* or neurotonin* or horizant*).mp. [mp=title, abstract, subject headings, heading word, drug trade name, original title, device manufacturer, drug manufacturer, device trade name, keyword]
5. 1 or 2 or 3 or 4
6. exp pain/
7. pain*.mp. [mp=title, abstract, subject headings, heading word, drug trade name, original title, device manufacturer, drug manufacturer, device trade name, keyword]
8. 6 or 7
9. 5 and 8
10. limit 9 to (human and (adult <18 to 64 years> or aged <65+ years>))

Science Citation Index Expanded (1900 to September 2015) (4492 hits)
#3 4,492 #2 AND #1
#2 445,898 TS=(pain*)
#1 75,431 TS=(gaba* or neurontin* or neurotonin* or horizant*)

Preliminary searches performed 12th April 2016

Total number of references identified: references
Number of duplicates removed: references
Number of references in final list: references
Number of new references: references

Batch name: 160412_J Wetterslev_GABA

Cochrane Central Register of Controlled Trials (CENTRAL) (Issue 4 of 12, 2015) (2993 hits)

#1 MeSH descriptor: [Amines] explode all trees and with qualifiers: [Adverse effects - AE, Therapeutic use - TU]
#2 MeSH descriptor: [gamma-Aminobutyric Acid] explode all trees and with qualifiers: [Adverse effects - AE, Therapeutic use - TU]
#3 MeSH descriptor: [Cyclohexanecarboxylic Acids] explode all trees and with qualifiers: [Adverse effects - AE, Therapeutic use - TU]
#4 (gaba* or neurontin* or neurotonin* or horizant*)
#5 #1 or #2 or #3 or #4
#6 MeSH descriptor: [Pain] explode all trees
#7 pain*
#8 #6 or #7
#9 #5 and #8
#10 adult* or middle age* or aged
#11 #9 and #10

<u>MEDLINE (Ovid SP) (1946 to April 2016) (6625 hits)</u>
1. exp Amines/ae, tu [Adverse Effects, Therapeutic Use]
2. exp gamma-Aminobutyric Acid/ae, tu [Adverse Effects, Therapeutic Use]
3. exp Cyclohexanecarboxylic Acids/ae, tu [Adverse Effects, Therapeutic Use]
4. (gaba* or neurontin* or neurotonin* or horizant*).mp. [mp=title, abstract, original title, name of substance word, subject heading word, keyword heading word, protocol supplementary concept, rare disease supplementary concept, unique identifier]
5. 1 or 2 or 3 or 4
6. exp Pain/
7. pain*.mp. [mp=title, abstract, original title, name of substance word, subject heading word, keyword heading word, protocol supplementary concept, rare disease supplementary concept, unique identifier]
8. 6 or 7
9. 5 and 8
10. limit 9 to (humans and ("all adult (19 plus years)" or "young adult (19 to 24 years)" or "adult (19 to 44 years)" or "young adult and adult (19-24 and 19-44)" or "middle age (45 to 64 years)" or "middle aged (45 plus years)" or "all aged (65 and over)" or "aged (80 and over)"))

<u>EMBASE (1974 to April 2016) (4474 hits)</u>
1. amine/ae, dt, th [Adverse Drug Reaction, Drug Therapy, Therapy]
2. 4 aminobutyric acid/ae, dt [Adverse Drug Reaction, Drug Therapy]
3. cyclohexanecarboxylic acid derivative/ae, dt [Adverse Drug Reaction, Drug Therapy]
4. (gaba* or neurontin* or neurotonin* or horizant*).mp. [mp=title, abstract, subject headings, heading word, drug trade name, original title, device manufacturer, drug manufacturer, device trade name, keyword]
5. 1 or 2 or 3 or 4
6. exp pain/
7. pain*.mp. [mp=title, abstract, subject headings, heading word, drug trade name, original title, device manufacturer, drug manufacturer, device trade name, keyword]
8. 6 or 7
9. 5 and 8
10. limit 9 to (human and (adult <18 to 64 years> or aged <65+ years>))

Science Citation Index Expanded (1900 to April 2016) (4717 hits)
#3 #2 AND #1
#2 TS=(pain*)
#1 TS=(gaba* or neurontin* or neurotonin* or horizant*)

Google Scholar search

After the 1st search, 13th November 2013
Gabapentin AND Postoperative pain
Gabapentin AND Acute pain management
Gabapentin AND Perioperative pain management

After the 2nd search, 30th June 2014
Gabapentin AND Postoperative pain
Gabapentin AND Acute pain management
Gabapentin AND Perioperative pain management

Limits: titles from 1st November 2013 and on

After the 3rd search, 14th November 2014
Gabapentin AND Postoperative pain
Gabapentin AND Acute pain management
Gabapentin AND Perioperative pain management

Limits: titles from 1st June 2014 and on

After the 4th search, 9th April 2015
Gabapentin AND Postoperative pain
Gabapentin AND Acute pain management
Gabapentin AND Perioperative pain management

Limits: titles from 1st November 2014 and on

After the 5th search, 23rd September 2015
Gabapentin AND Postoperative pain
Gabapentin AND Acute pain management
Gabapentin AND Perioperative pain management

Limits: titles from 1st April 2015 and on

After the 6th search, 12th April 2016
Gabapentin AND Postoperative pain
Gabapentin AND Acute pain management
Gabapentin AND Perioperative pain management

Limits: titles from 1st September 2015 and on

Appendix 2: Opioid conversion table

**Opioid**	**Administration**	**Opioid: Intravenous morphine**
1 mg Fentanyl	i.v.	100 mg morphine
1 mg Hydromorphone	i.v.	5 mg morphine
1 mg Morphine oral	oral	0.33 mg morphine
1 mg Nalbuphine	i.v.	1 mg morphine
1 mg Pethidine/Meperidine	i.v.	0.13 mg morphine
1 mg Propoxyphene	i.v.	5 mg morphine
1 mg Tramadol oral	oral	0.07 mg morphine

Appendix 3: Forest plot of 24-hour morphine consumption from all trials estimates

	Experimental			Control				Mean Difference
Study or Subgroup	Mean [mg]	SD [mg]	Total	Mean [mg]	SD [mg]	Total	Weight	IV, Random, 95% CI [mg]
**1.1.1 Cholecystectomy**								
Bekawi 2014	0	2.2	30	7.5	0.7	32	2.6%	-7.50 [-8.32, -6.68]
Hoseini 2015	65.11	11.81	22	78.41	13.3	22	1.7%	-13.30 [-20.73, -5.87]
Khademi 2009	2.83	1.29	44	3.51	1.51	43	2.6%	-0.68 [-1.27, -0.09]
Maleh 2013	2.5	2.6	40	2.7	2.7	40	2.5%	-0.20 [-1.36, 0.96]
Mishra 2016	8.1	1	30	11.9	1.4	30	2.6%	-3.80 [-4.42, -3.18]
Panday 2004b	40.42	31.26	153	53.03	27.48	153	1.8%	-12.61 [-19.21, -6.01]
Panday 2006	39.19	26.31	125	67.66	25.27	125	1.9%	-28.47 [-34.87, -22.07]
Srivastava 2010	25.39	4.48	60	37.58	8.35	60	2.4%	-12.19 [-14.59, -9.79]
Syal 2010	40.2	35.2	30	46.7	35.8	30	0.6%	-6.50 [-24.47, 11.47]
Takmaz 2007	5.47	3.43	30	6.25	3.44	15	2.5%	-0.78 [-2.91, 1.35]
**Subtotal (95% CI)**			564			550	21.1%	-7.25 [-9.94, -4.55]
Heterogeneity: Tau^2 = 14.30; Chi^2 = 337.70, df = 9 (P < 0.00001); I^2 = 97%								
Test for overall effect: Z = 5.27 (P < 0.00001)								
**1.1.2 Hysterectomy**								
Badawy 2014	11.5	2.3	19	13	2.9	19	2.5%	-1.50 [-3.16, 0.16]
Dierking 2003	43	23.7	39	63	25.9	32	1.1%	-20.00 [-31.66, -8.34]
Durmus 2007	40	10	25	66	10	25	2.0%	-26.00 [-31.54, -20.46]
Fassoulaki 2005	20.3	7.9	25	25.7	11.2	28	2.1%	-5.40 [-10.58, -0.22]
Fassoulaki 2006	22	2.9	27	35	4.8	24	2.5%	-13.00 [-15.21, -10.79]
Frouzanfard 2013	1.2	0.29	25	5.2	2.8	25	2.5%	-4.00 [-5.10, -2.90]
Ghafari 2009	15.78	1.15	33	26.94	2.28	33	2.6%	-11.16 [-12.03, -10.29]
Ghai 2011	5.44	1.56	30	4.28	1.87	30	2.6%	1.16 [0.29, 2.03]
Gilron 2004	56.78	32.41	23	82.11	48.2	24	0.4%	-25.33 [-48.72, -1.94]
Joseph 2014	38.65	18.04	25	44.29	16.02	25	1.4%	-5.64 [-15.10, 3.82]
Khan 2013	13.21	4.71	34	24.31	9.28	35	2.3%	-11.10 [-14.56, -7.64]
Sekhavet 2009	40.1	14.5	49	52.7	21.1	49	1.7%	-12.60 [-19.77, -5.43]
Sen 2009a	31	12	20	48	17	20	1.4%	-17.00 [-26.12, -7.88]
Turan 2003a	27.04	14.44	25	41.96	8.36	25	1.8%	-14.92 [-21.46, -8.38]
**Subtotal (95% CI)**			399			394	26.9%	-10.53 [-14.38, -6.69]
Heterogeneity: Tau^2 = 43.15; Chi^2 = 548.76, df = 13 (P < 0.00001); I^2 = 98%								
Test for overall effect: Z = 5.37 (P < 0.00001)								
**1.1.3 Mastectomy**								
Amr 2009	13.5	0.5	50	22	2.1	50	2.6%	-8.50 [-9.10, -7.90]
Bharti 2012	2.1	2.2	20	4.9	3.4	20	2.5%	-2.80 [-4.57, -1.03]
Doha 2010	39.9	33	30	42.7	36.1	29	0.6%	-2.80 [-20.47, 14.87]
Fassoulaki 2002	23.8	5	25	23.2	5.8	25	2.4%	0.60 [-2.40, 3.60]
Kim 2004	35.8	20.8	21	33.5	26.1	20	0.9%	2.30 [-12.19, 16.79]
Metry 2008	16.1	7.7	67	29.2	9.6	34	2.3%	-13.10 [-16.82, -9.38]
**Subtotal (95% CI)**			213			178	11.2%	-5.19 [-9.52, -0.86]
Heterogeneity: Tau^2 = 20.39; Chi^2 = 75.74, df = 5 (P < 0.00001); I^2 = 93%								
Test for overall effect: Z = 2.35 (P = 0.02)								
**1.1.4 Orthopaedic surgery**								
Clarke 2009a	33.2	20.73	29	63.8	36.5	7	0.3%	-30.60 [-58.67, -2.53]
Clarke 2009b	33.1	4	76	35	4	39	2.5%	-1.90 [-3.44, -0.36]
Clarke 2014	37	1.5	95	48	1.33	84	2.6%	-11.00 [-11.41, -10.59]
Lunn 2015	42.8	38.4	186	50.5	41.4	99	1.3%	-7.70 [-17.55, 2.15]
Paul 2013	27.94	22.99	52	26.77	18.96	49	1.6%	1.17 [-7.03, 9.37]
Paul 2015	19.7	16.39	48	25.1	14.5	54	1.9%	-5.40 [-11.44, 0.64]
**Subtotal (95% CI)**			486			332	10.2%	-6.11 [-12.05, -0.18]
Heterogeneity: Tau^2 = 38.90; Chi^2 = 136.90, df = 5 (P < 0.00001); I^2 = 96%								
Test for overall effect: Z = 2.02 (P = 0.04)								
**1.1.5 Spinal Surgery**								
Erten 2010	3.31	0.98	39	3.56	0.64	20	2.6%	-0.25 [-0.67, 0.17]
Khan 2010	20.8	5.72	150	31.5	9.6	25	2.3%	-10.70 [-14.57, -6.83]
Panday 2004a	39.18	22.31	80	52.65	21.27	20	1.3%	-13.47 [-24.00, -2.94]
Panday 2004c	90.85	34.1	28	92.49	41.77	28	0.5%	-1.64 [-21.61, 18.33]
Turan 2003b	16.3	8.9	25	42.8	10.9	25	2.0%	-26.50 [-32.02, -20.98]
Vahedi 2011	18.61	9.03	36	21.53	11.3	40	2.1%	-2.92 [-7.50, 1.66]
Vasigh 2016	11.9	4.4	38	30.1	0.6	38	2.5%	-18.20 [-19.61, -16.79]
Özgencil 2011	29.47	9.64	30	37.33	9.5	30	2.1%	-7.86 [-12.70, -3.02]
**Subtotal (95% CI)**			426			226	15.4%	-10.55 [-19.04, -2.05]
Heterogeneity: Tau^2 = 135.70; Chi^2 = 679.10, df = 7 (P < 0.00001); I^2 = 99%								
Test for overall effect: Z = 2.43 (P = 0.01)								
**1.1.6 Thoracic surgery**								
Grosen 2014	11.2	21.62	52	17.92	23.55	52	1.5%	-6.72 [-15.41, 1.97]
Hout 2007	2.36	2.5	23	2.65	3.2	28	2.5%	-0.29 [-1.85, 1.27]
Kosucu 2013	25.9	8.3	29	44	11	31	2.1%	-18.10 [-23.01, -13.19]
Menda 2010	6	8.5	30	15.1	20	30	1.6%	-9.10 [-16.88, -1.32]
Omran 2006	23.9	2.6	25	31.5	2.78	25	2.5%	-7.60 [-9.09, -6.11]
Soltanzadeh 2011	2.5	0.9	30	4	1.5	30	2.6%	-1.50 [-2.13, -0.87]
Ucak 2011	9.9	5.38	20	14.94	7.25	20	2.2%	-5.04 [-9.00, -1.08]
**Subtotal (95% CI)**			209			216	15.1%	-6.27 [-9.67, -2.86]
Heterogeneity: Tau^2 = 16.31; Chi^2 = 106.32, df = 6 (P < 0.00001); I^2 = 94%								
Test for overall effect: Z = 3.61 (P = 0.0003)								
**Total (95% CI)**			2297			1896	100.0%	-8.20 [-9.85, -6.56]
Heterogeneity: Tau^2 = 27.42; Chi^2 = 3081.02, df = 50 (P < 0.00001); I^2 = 98%								
Test for overall effect: Z = 9.76 (P < 0.00001)								
Test for subgroup differences: Chi^2 = 4.70, df = 5 (P = 0.45), I^2 = 0%								

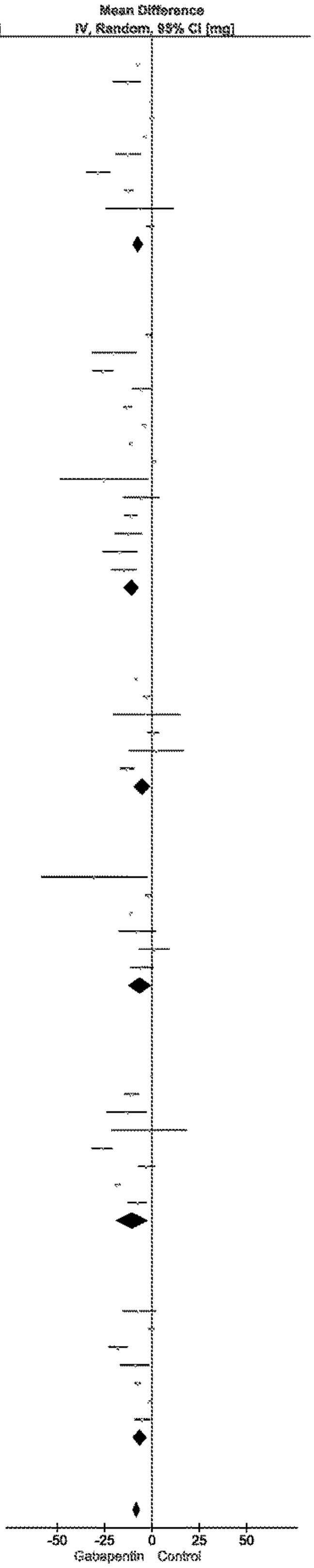

Appendix 4: TSA of 24-hour morphine consumption in the cholecystectomy subgroup, all trials

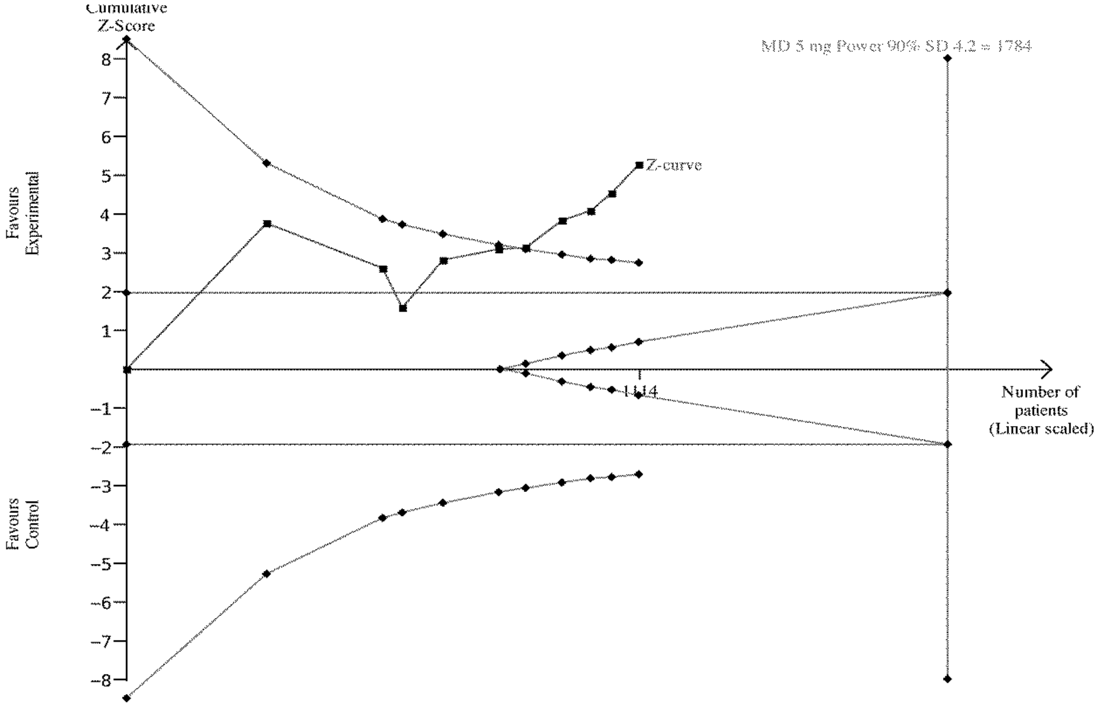

Appendix 5: TSA of 24-hour morphine consumption in the hysterectomy subgroup, all trials

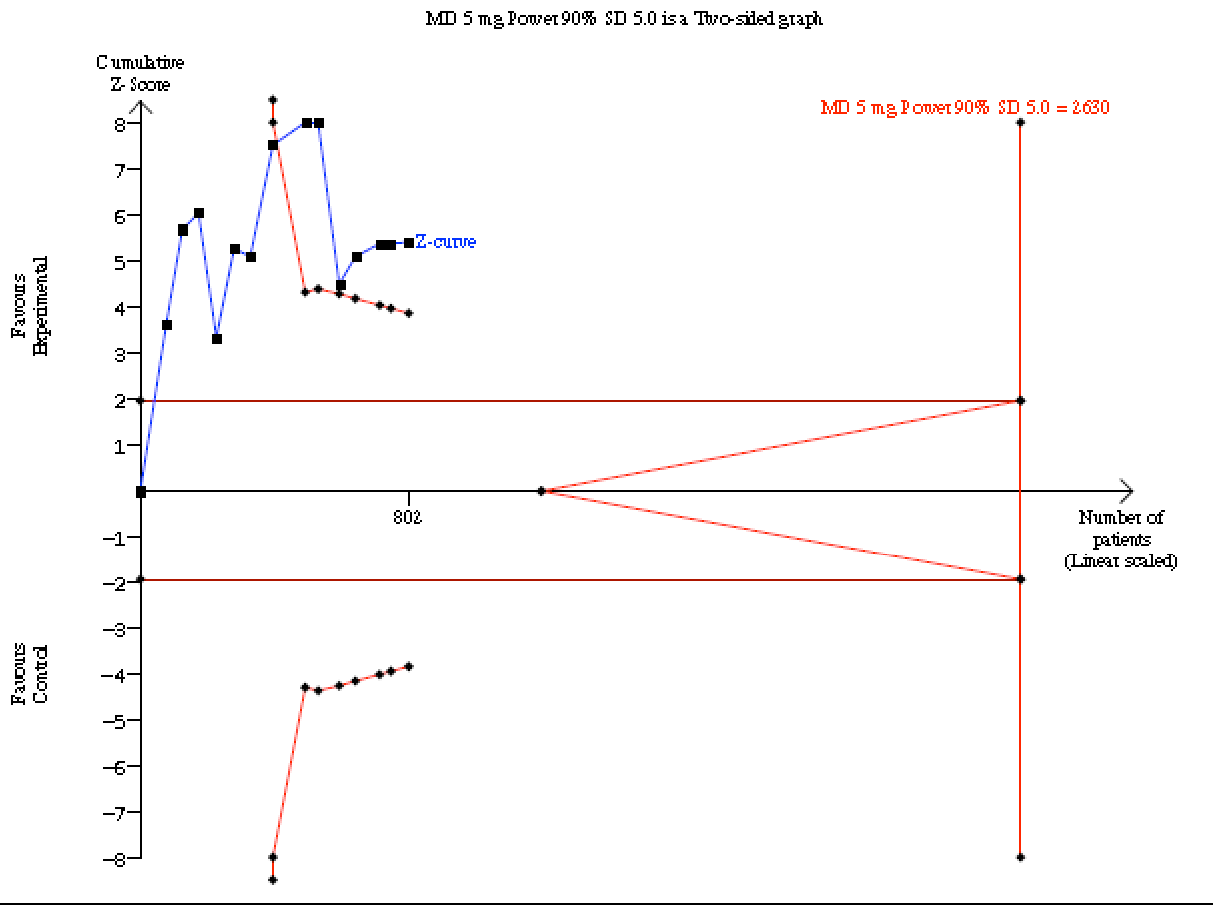

Appendix 6: TSA of 24-hour morphine consumption in the mastectomy subgroup, all trials

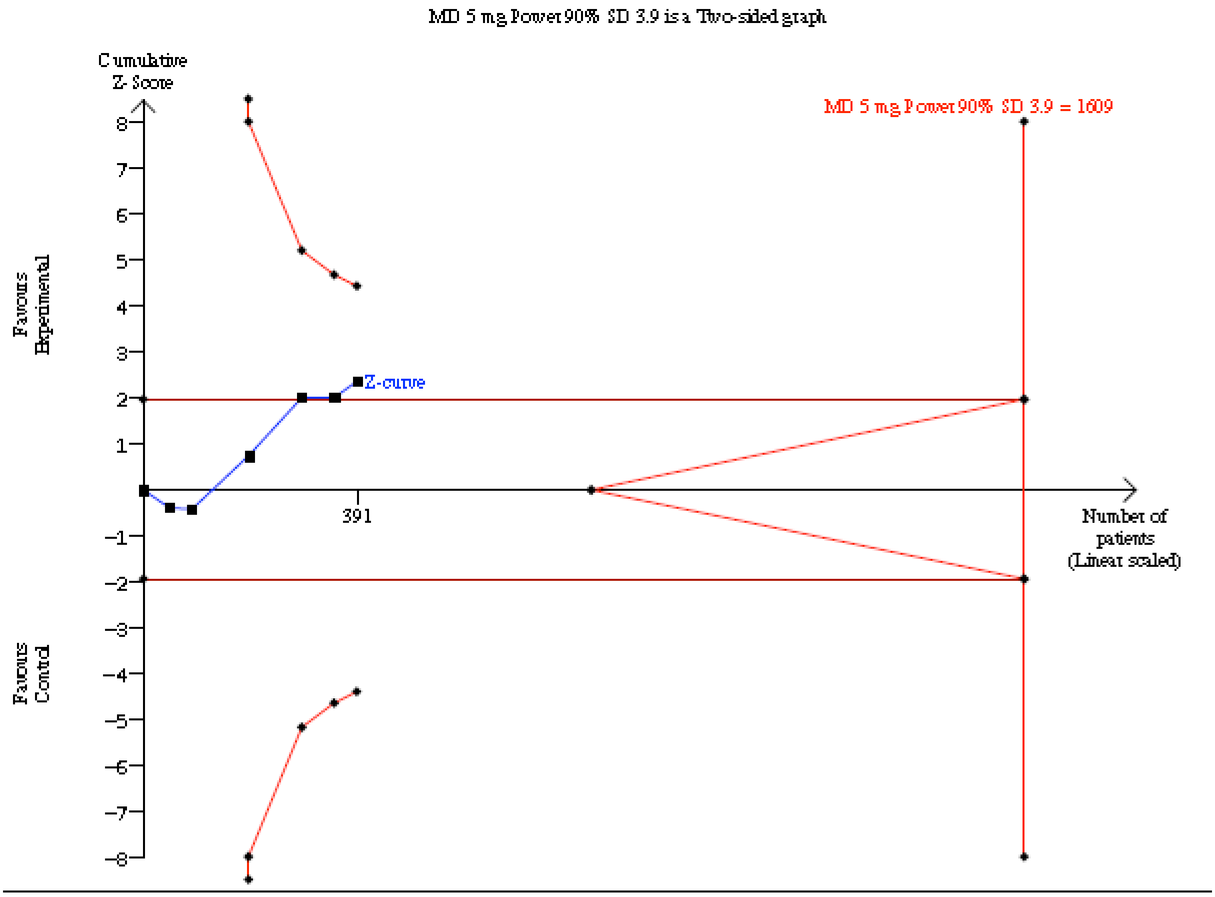

Appendix 7: TSA of 24-hour morphine consumption in the orthopedic arthroplasty subgroup, all trials

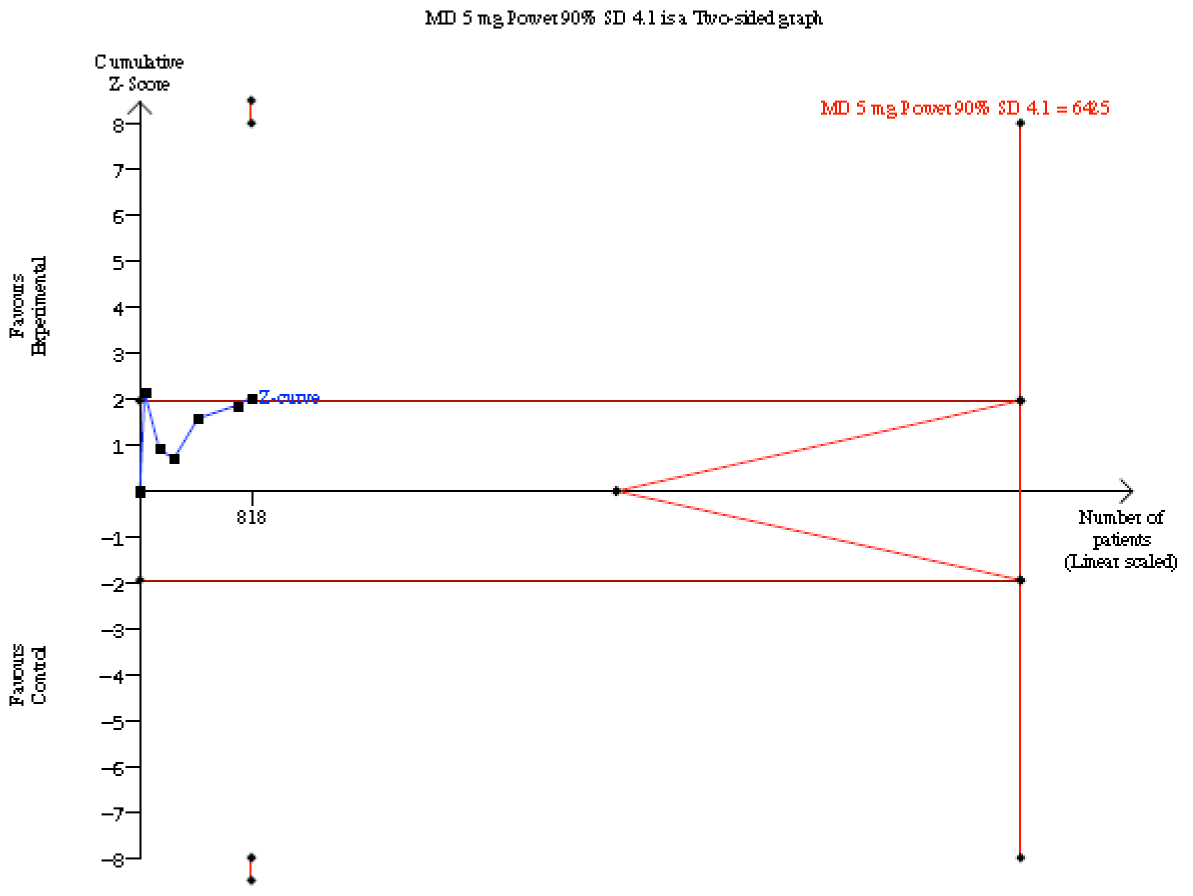

Appendix 8: TSA of 24-hour morphine consumption in the spinal subgroup, all trials

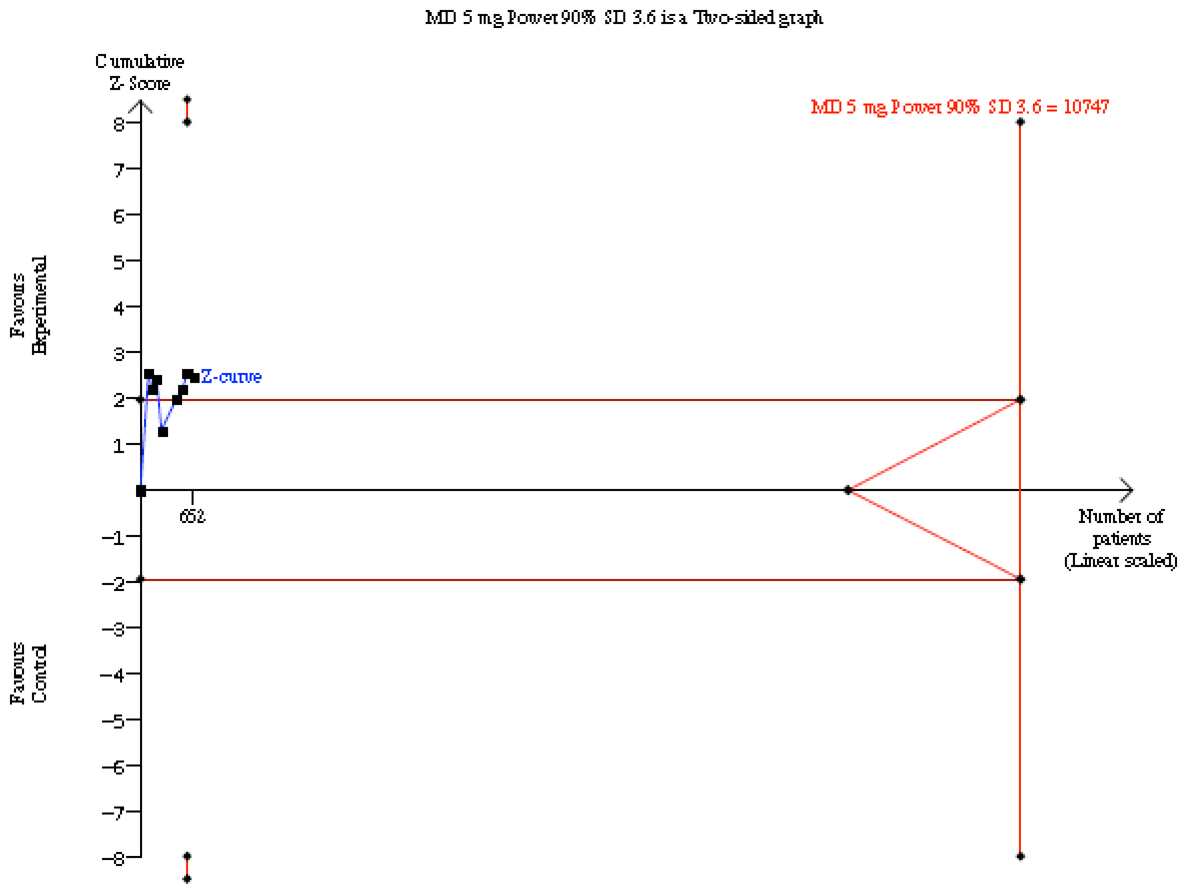

Appendix 9: TSA of 24-hour morphine consumption in the thoracic surgery subgroup, all trials

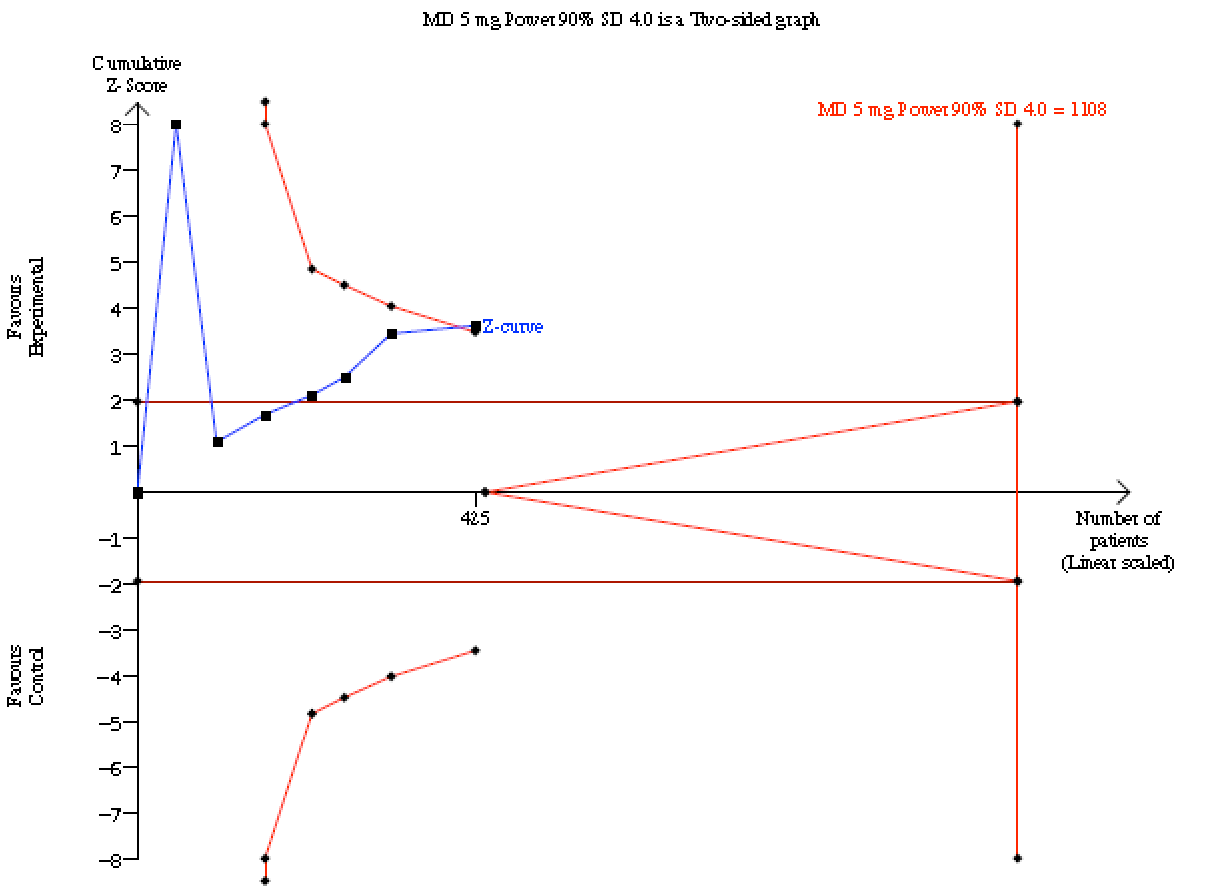

Appendix 10: Forest plot of SAEs from all trials

	Gabapentin		Control			Risk Ratio
Study or Subgroup	Events	Total	Events	Total	Weight	M-H, Fixed, 95% CI
**1.14.1 Cholecystectomy**						
Srivastava 2010	0	60	0	60		Not estimable
**Subtotal (95% CI)**		**60**		**60**		**Not estimable**
Total events	0		0			
Heterogeneity: Not applicable						
Test for overall effect: Not applicable						
**1.14.2 Hysterectomy**						
Ajori 2011	0	69	0	69		Not estimable
Dierking 2003	0	39	0	32		Not estimable
Fassoulaki 2006	0	27	0	24		Not estimable
Gilron 2004	1	20	2	22	6.9%	0.55 [0.05, 5.61]
Khan 2013	0	34	0	35		Not estimable
**Subtotal (95% CI)**		**189**		**182**	**6.9%**	**0.55 [0.05, 5.61]**
Total events	1		2			
Heterogeneity: Not applicable						
Test for overall effect: Z = 0.50 (P = 0.61)						
**1.14.3 Mastectomy**						
Dirks 2002	0	31	0	34		Not estimable
Fassoulaki 2002	0	25	0	25		Not estimable
**Subtotal (95% CI)**		**56**		**59**		**Not estimable**
Total events	0		0			
Heterogeneity: Not applicable						
Test for overall effect: Not applicable						
**1.14.4 Orthopaedic surgery**						
Lunn 2015	6	183	1	91	4.9%	2.98 [0.36, 24.41]
Paul 2013	0	52	0	49		Not estimable
**Subtotal (95% CI)**		**235**		**140**	**4.9%**	**2.98 [0.36, 24.41]**
Total events	6		1			
Heterogeneity: Not applicable						
Test for overall effect: Z = 1.02 (P = 0.31)						
**1.14.5 Spinal surgery**						
Vahedi 2011	0	36	0	40		Not estimable
**Subtotal (95% CI)**		**36**		**40**		**Not estimable**
Total events	0		0			
Heterogeneity: Not applicable						
Test for overall effect: Not applicable						
**1.14.6 Thoracic surgery**						
Grosen 2014	13	52	8	52	29.1%	1.63 [0.74, 3.59]
Hout 2007	1	28	0	28	1.8%	3.00 [0.13, 70.64]
Kinney 2011	4	57	5	63	17.3%	0.88 [0.25, 3.13]
Ucak 2011	7	20	11	20	40.0%	0.64 [0.31, 1.30]
**Subtotal (95% CI)**		**157**		**163**	**88.2%**	**1.06 [0.66, 1.71]**
Total events	25		24			
Heterogeneity: Chi² = 3.56, df = 3 (P = 0.31); I² = 16%						
Test for overall effect: Z = 0.24 (P = 0.81)						
**Total (95% CI)**		**733**		**644**	**100.0%**	**1.12 [0.71, 1.77]**
Total events	32		27			
Heterogeneity: Chi² = 4.93, df = 5 (P = 0.42); I² = 0%						
Test for overall effect: Z = 0.48 (P = 0.63)						
Test for subgroup differences: Chi² = 1.23, df = 2 (P = 0.54), I² = 0%						

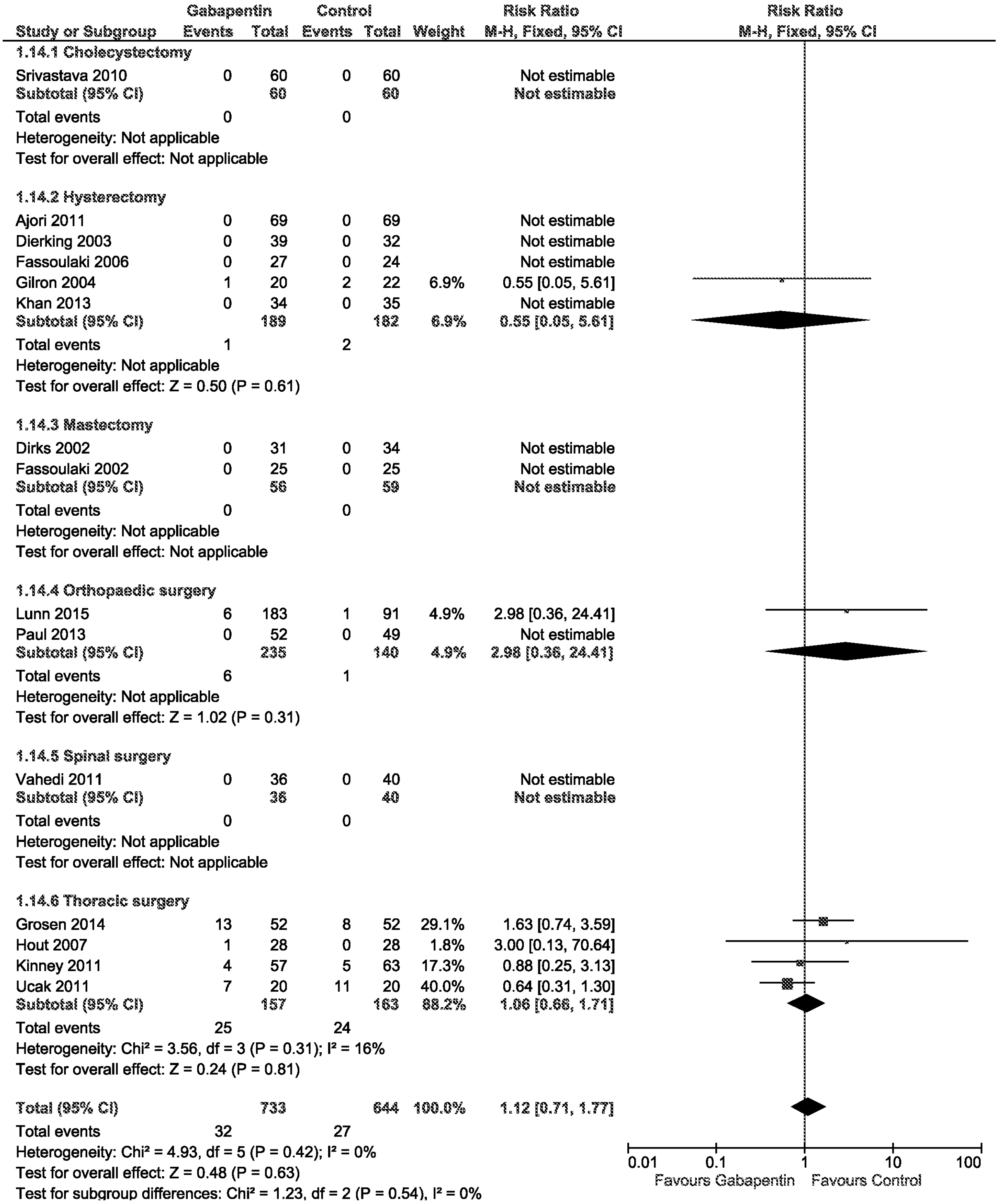

Appendix 11: TSA of SAEs in the thoracic surgery subgroup, all trials

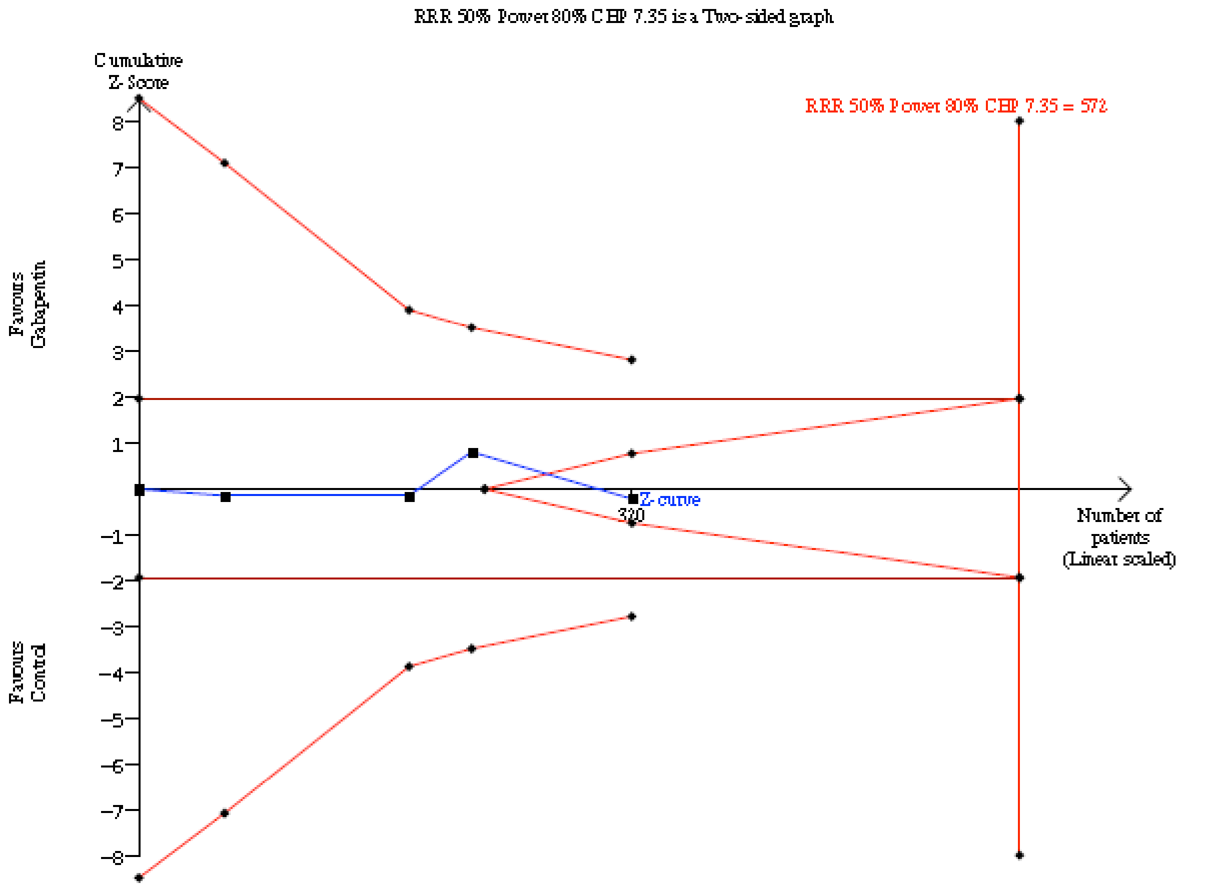

Appendix 12: Forest plot of VAS 6 hours at rest, all trials

	Gabapentin			Control				Mean Difference
Study or Subgroup	Mean [VAS]	SD [VAS]	Total	Mean [VAS]	SD [VAS]	Total	Weight	IV, Random, 95% CI [VAS]
**2.1.1 Cholecystectomy**								
Hoseini 2015	16.4	10.2	22	30.9	10.1	22	2.8%	-14.50 [-20.50, -8.50]
Mishra 2016	30.7	4.4	30	31	2.1	30	3.0%	-0.30 [-2.04, 1.44]
Srivastava 2010	23	3.17	60	49	3.33	60	3.0%	-26.00 [-27.16, -24.84]
**Subtotal (95% CI)**			**112**			**112**	**8.7%**	**-13.60 [-33.36, 6.17]**
Heterogeneity: Tau² = 301.60; Chi² = 578.37, df = 2 (P < 0.00001); I² = 100%								
Test for overall effect: Z = 1.35 (P = 0.18)								
**2.1.2 Hysterectomy**								
Ajori 2011	40	30	69	63	28	69	2.5%	-23.00 [-32.68, -13.32]
Badawy 2014	40	5	19	45	5	19	2.9%	-5.00 [-8.18, -1.82]
Behdad 2012	38	9.6	30	66.2	13.7	31	2.8%	-28.20 [-34.12, -22.28]
Dierking 2003	14.12	15.23	40	21.53	18.59	40	2.7%	-7.41 [-14.86, 0.04]
Durmus 2007	45	51	25	52	61	25	0.9%	-7.00 [-38.17, 24.17]
Fassoulaki 2005	17	28	25	20	31	28	1.9%	-3.00 [-18.88, 12.88]
Fassoulaki 2006	25.7	16.1	27	26.8	19.6	24	2.4%	-1.10 [-11.02, 8.82]
Frouzanfard 2013	35.6	15	25	65.6	18	25	2.5%	-30.00 [-39.18, -20.82]
Ghafari 2009	42.5	3.5	33	58.1	4	33	3.0%	-15.60 [-17.41, -13.79]
Gilron 2004	29	4	23	39	3	24	3.0%	-10.00 [-12.03, -7.97]
Khan 2013	36.17	13.4	34	52	10.51	35	2.8%	-15.83 [-21.52, -10.14]
Ray 2015	56.16	14.24	30	77	12.9	30	2.7%	-20.84 [-27.72, -13.96]
Sekhavet 2009	37.9	20.8	49	76.6	22.4	49	2.6%	-38.70 [-47.26, -30.14]
Sen 2009a	28	24	20	41	47	20	1.4%	-13.00 [-36.13, 10.13]
Turan 2003a	18	16	25	42	10	25	2.7%	-24.00 [-31.40, -16.60]
Verma 2008	23	14	25	32	16	25	2.6%	-9.00 [-17.33, -0.67]
**Subtotal (95% CI)**			**499**			**502**	**39.2%**	**-16.43 [-20.62, -12.24]**
Heterogeneity: Tau² = 52.07; Chi² = 141.40, df = 15 (P < 0.00001); I² = 89%								
Test for overall effect: Z = 7.69 (P < 0.00001)								
**2.1.3 Mastectomy**								
Dirks 2002	14	18	31	18	15	34	2.6%	-4.00 [-12.10, 4.10]
Doha 2010	18	17	30	33	11	29	2.7%	-15.00 [-22.28, -7.72]
Fassoulaki 2002	7	20	25	9	19	25	2.4%	-2.00 [-12.81, 8.81]
Gosai 2015	36	5	30	52	8	30	2.9%	-16.00 [-19.38, -12.62]
Grover 2009	10	8.15	27	10	8.15	23	2.9%	0.00 [-4.53, 4.53]
Kim 2004	27	22.2	21	29	17.6	20	2.2%	-2.00 [-14.23, 10.23]
Metry 2008	12.52	9.5	67	24.1	13	34	2.8%	-11.58 [-16.51, -6.65]
**Subtotal (95% CI)**			**231**			**195**	**18.5%**	**-7.80 [-13.73, -1.87]**
Heterogeneity: Tau² = 49.77; Chi² = 38.89, df = 6 (P < 0.00001); I² = 85%								
Test for overall effect: Z = 2.58 (P = 0.010)								
**2.1.4 Orthopaedic surgery**								
**Subtotal (95% CI)**			**0**			**0**		**Not estimable**
Heterogeneity: Not applicable								
Test for overall effect: Not applicable								
**2.1.5 Spinal surgery**								
Erten 2010	25.89	7.11	39	30.5	8.8	20	2.9%	-4.61 [-9.07, -0.15]
Khan 2010	44.4	14.55	150	68	11	25	2.8%	-23.60 [-28.50, -18.70]
Panday 2004a	36.5	12.97	80	61.5	13	20	2.7%	-25.00 [-31.37, -18.63]
Radhakrishnan 2005	10	12.5	30	10	12.5	30	2.7%	0.00 [-6.33, 6.33]
Turan 2003b	13	15	25	24	18	25	2.5%	-11.00 [-20.18, -1.82]
Vahedi 2011	61.1	20.9	36	56.8	24.4	40	2.4%	4.30 [-5.89, 14.49]
Özgencil 2011	24	6.7	30	33.3	10.9	30	2.9%	-9.30 [-13.88, -4.72]
**Subtotal (95% CI)**			**390**			**190**	**19.0%**	**-10.17 [-18.02, -2.32]**
Heterogeneity: Tau² = 100.36; Chi² = 72.64, df = 6 (P < 0.00001); I² = 92%								
Test for overall effect: Z = 2.54 (P = 0.01)								
**2.1.6 Thoracic surgery**								
Grosen 2014	6.84	12.97	52	13.95	17.94	52	2.8%	-7.11 [-13.13, -1.09]
Hout 2007	0	5.93	23	0	3.7	28	2.9%	0.00 [-2.78, 2.78]
Kinney 2011	27	25.2	57	28	26.2	68	2.5%	-1.00 [-10.03, 8.03]
Kosucu 2013	29	12	29	42	21	31	2.6%	-13.00 [-21.59, -4.41]
Menda 2010	20	31	30	30	48	30	1.5%	-10.00 [-30.45, 10.45]
Omran 2006	35	45.59	25	61	45.69	25	1.2%	-26.00 [-51.30, -0.70]
Ucak 2011	29.9	2	20	50	65	20	1.1%	-20.10 [-48.60, 8.40]
**Subtotal (95% CI)**			**236**			**254**	**14.6%**	**-6.63 [-12.42, -0.84]**
Heterogeneity: Tau² = 29.37; Chi² = 16.50, df = 6 (P = 0.01); I² = 64%								
Test for overall effect: Z = 2.24 (P = 0.02)								
**Total (95% CI)**			**1468**			**1253**	**100.0%**	**-12.14 [-15.80, -8.48]**
Heterogeneity: Tau² = 116.58; Chi² = 1030.96, df = 39 (P < 0.00001); I² = 96%								
Test for overall effect: Z = 6.51 (P < 0.00001)								
Test for subgroup differences: Chi² = 9.68, df = 4 (P = 0.05), I² = 58.7%								

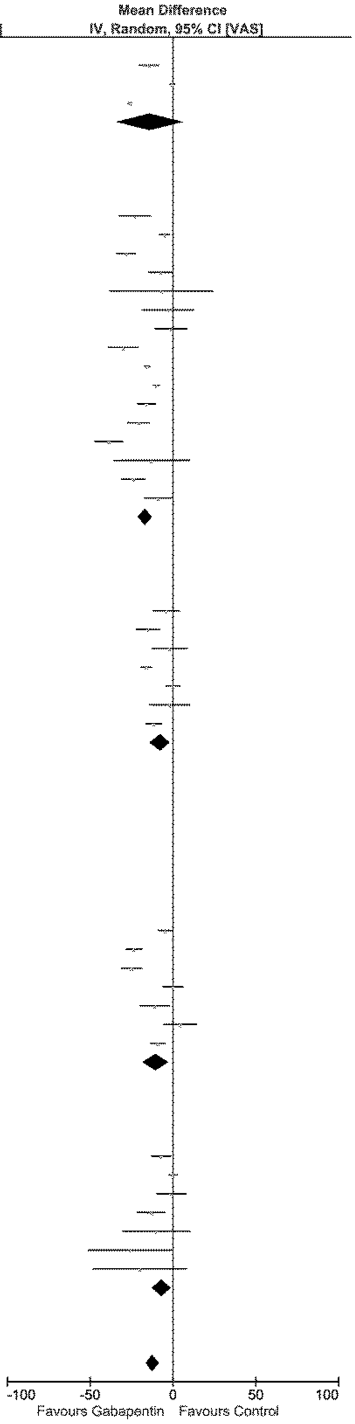

Appendix 13: Forest plot of VAS 6 hours at mobilization, all trials

	Gabapentin			Control				Mean Difference	Mean Difference
Study or Subgroup	Mean [VAS]	SD [VAS]	Total	Mean [VAS]	SD [VAS]	Total	Weight	IV, Random, 95% CI [VAS]	IV, Random, 95% CI [VAS]
**2.2.1 Cholecystectomy**									
Srivastava 2010	59	3.33	60	70.5	3.5	60	22.5%	-11.50 [-12.72, -10.28]	
**Subtotal (95% CI)**			**60**			**60**	**22.5%**	**-11.50 [-12.72, -10.28]**	
Heterogeneity: Not applicable									
Test for overall effect: Z = 18.44 (P < 0.00001)									
**2.2.2 Hysterectomy**									
Dierking 2003	42.53	25.9	40	51.09	28.27	40	5.1%	-8.56 [-20.44, 3.32]	
Durmus 2007	67	77	25	76	83	25	0.5%	-9.00 [-53.38, 35.38]	
Fassoulaki 2005	40	67	25	53	78	28	0.6%	-13.00 [-52.04, 26.04]	
Fassoulaki 2006	47.8	19.7	27	45.1	24.6	24	4.8%	2.70 [-9.63, 15.03]	
Sen 2009a	32	34	20	56	54	20	1.1%	-24.00 [-51.97, 3.97]	
Turan 2003a	31.2	17	25	43	12	25	8.7%	-11.80 [-19.96, -3.64]	
**Subtotal (95% CI)**			**162**			**162**	**20.7%**	**-8.47 [-14.18, -2.76]**	
Heterogeneity: Tau² = 0.36; Chi² = 5.03, df = 5 (P = 0.41); I² = 1%									
Test for overall effect: Z = 2.91 (P = 0.004)									
**2.2.3 Mastectomy**									
Dirks 2002	19	21	31	31	23	34	6.0%	-12.00 [-22.70, -1.30]	
Fassoulaki 2002	21	35	25	29	52	25	1.4%	-8.00 [-32.57, 16.57]	
Grover 2009	30	14.81	27	30	7.41	23	11.5%	0.00 [-6.35, 6.35]	
Kim 2004	42	25.2	21	37	15.8	20	4.5%	5.00 [-7.81, 17.81]	
Metry 2008	20.52	14.48	67	31.3	15	34	11.9%	-10.78 [-16.90, -4.66]	
**Subtotal (95% CI)**			**171**			**136**	**35.4%**	**-5.20 [-12.04, 1.65]**	
Heterogeneity: Tau² = 32.18; Chi² = 9.78, df = 4 (P = 0.04); I² = 59%									
Test for overall effect: Z = 1.49 (P = 0.14)									
**2.2.4 Orthopaedic surgery**									
**Subtotal (95% CI)**			**0**			**0**		**Not estimable**	
Heterogeneity: Not applicable									
Test for overall effect: Not applicable									
**2.2.5 Spinal surgery**									
Radhakrishnan 2005	20	15	30	30	20	30	7.7%	-10.00 [-18.95, -1.05]	
**Subtotal (95% CI)**			**30**			**30**	**7.7%**	**-10.00 [-18.95, -1.05]**	
Heterogeneity: Not applicable									
Test for overall effect: Z = 2.19 (P = 0.03)									
**2.2.6 Thoracic surgery**									
Grosen 2014	12.42	18.55	52	21.29	19.28	52	9.9%	-8.87 [-16.14, -1.60]	
Menda 2010	43.5	57	30	65	78	30	0.7%	-21.50 [-56.07, 13.07]	
Omran 2006	60	35.16	25	80	35.16	25	2.2%	-20.00 [-39.49, -0.51]	
Ucak 2011	39	20	20	55	68	20	0.9%	-16.00 [-47.06, 15.06]	
**Subtotal (95% CI)**			**127**			**127**	**13.8%**	**-10.89 [-17.42, -4.35]**	
Heterogeneity: Tau² = 0.00; Chi² = 1.60, df = 3 (P = 0.66); I² = 0%									
Test for overall effect: Z = 3.27 (P = 0.001)									
**Total (95% CI)**			**550**			**515**	**100.0%**	**-8.58 [-11.61, -5.56]**	
Heterogeneity: Tau² = 10.22; Chi² = 25.60, df = 16 (P = 0.06); I² = 37%									
Test for overall effect: Z = 5.56 (P < 0.00001)									
Test for subgroup differences: Chi² = 4.14, df = 4 (P = 0.39), I² = 3.4%									

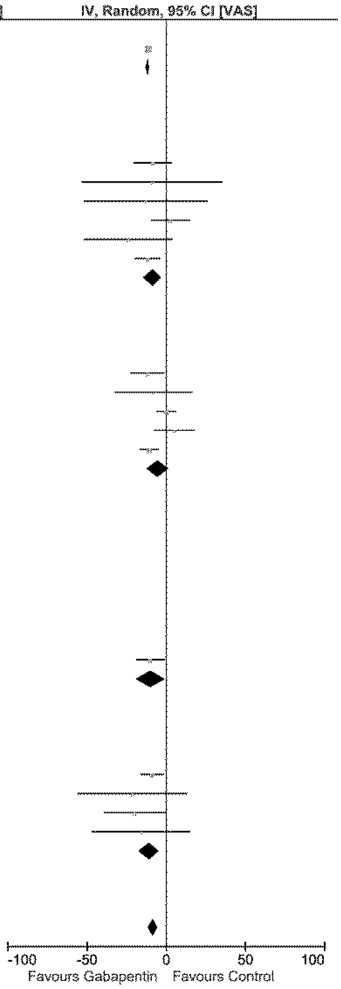

Appendix 14: Forest plot of VAS 24 hours at rest, all trials

	Gabapentin			Control				Mean Difference
Study or Subgroup	Mean [VAS]	SD [VAS]	Total	Mean [VAS]	SD [VAS]	Total	Weight	IV, Random, 95% CI [VAS]
**2.3.1 Cholecystectomy**								
Bekawi 2014	9.7	5.6	30	6	6.2	32	3.7%	3.70 [0.76, 6.64]
Mishra 2016	20.1	3.4	30	26	4.1	30	3.9%	-5.90 [-7.81, -3.99]
Srivastava 2010	21	3.25	60	28	3.33	60	4.0%	-7.00 [-8.18, -5.82]
**Subtotal (95% CI)**			**120**			**122**	**11.6%**	**-3.24 [-8.37, 1.88]**
Heterogeneity: Tau² = 19.38; Chi² = 44.02, df = 2 (P < 0.00001); I² = 95%								
Test for overall effect: Z = 1.24 (P = 0.22)								
**2.3.2 Hysterectomy**								
Ajori 2011	2	8	69	9	13	69	3.6%	-7.00 [-10.60, -3.40]
Badawy 2014	20	2.5	19	25	2.5	19	3.9%	-5.00 [-6.59, -3.41]
Behdad 2012	25.3	5	30	42.7	14.2	31	3.3%	-17.40 [-22.71, -12.09]
Dierking 2003	13.64	19.19	40	15.21	18.93	40	2.6%	-1.57 [-9.92, 6.78]
Durmus 2007	27	40	25	39	49	25	0.7%	-12.00 [-36.79, 12.79]
Fassoulaki 2005	16	27	25	7	14	28	1.9%	9.00 [-2.79, 20.79]
Frouzanfard 2013	5.6	5.8	25	17.2	12.7	25	3.2%	-11.60 [-17.07, -6.13]
Ghafari 2009	18.1	3	33	34.8	4	33	3.9%	-16.70 [-18.41, -14.99]
Gilron 2004	13.82	12.5	23	23.63	16.52	24	2.6%	-9.81 [-18.16, -1.46]
Khan 2013	8.52	7.43	34	24.28	11.18	35	3.4%	-15.76 [-20.23, -11.29]
Ray 2015	37	12.56	30	54.66	15.5	30	2.8%	-17.66 [-24.80, -10.52]
Sekhavet 2009	40.1	14.5	49	52.7	21.1	49	2.8%	-12.60 [-19.77, -5.43]
Sen 2009a	2	5	20	16	28	20	1.8%	-14.00 [-26.47, -1.53]
Turan 2003a	5	7	25	16	12	25	3.2%	-11.00 [-16.45, -5.55]
Verma 2008	12	13	25	21	12	25	2.9%	-9.00 [-15.94, -2.06]
**Subtotal (95% CI)**			**472**			**478**	**42.5%**	**-10.54 [-14.18, -6.89]**
Heterogeneity: Tau² = 38.17; Chi² = 131.90, df = 14 (P < 0.00001); I² = 89%								
Test for overall effect: Z = 5.67 (P < 0.00001)								
**2.3.3 Mastectomy**								
Fassoulaki 2002	4	12	25	7	14	25	2.8%	-3.00 [-10.23, 4.23]
Kim 2004	23	19	21	20	10.3	20	2.3%	3.00 [-6.30, 12.30]
Metry 2008	18.49	14.94	67	23	13	34	3.2%	-4.51 [-10.16, 1.14]
**Subtotal (95% CI)**			**113**			**79**	**8.3%**	**-2.64 [-6.66, 1.37]**
Heterogeneity: Tau² = 0.00; Chi² = 1.84, df = 2 (P = 0.40); I² = 0%								
Test for overall effect: Z = 1.29 (P = 0.20)								
**2.3.4 Orthopaedic surgery**								
Clarke 2009a	36.53	25.97	29	51	21.8	7	1.0%	-14.47 [-33.18, 4.24]
Clarke 2009b	13	3.97	76	14	4	39	3.9%	-1.00 [-2.54, 0.54]
Paul 2015	24.5	16.1	48	23.6	20.8	54	2.8%	0.90 [-6.28, 8.08]
**Subtotal (95% CI)**			**153**			**100**	**7.8%**	**-1.04 [-3.73, 1.66]**
Heterogeneity: Tau² = 1.59; Chi² = 2.26, df = 2 (P = 0.32); I² = 11%								
Test for overall effect: Z = 0.75 (P = 0.45)								
**2.3.5 Spinal surgery**								
Erten 2010	9.22	8.04	39	13	9.7	20	3.3%	-3.78 [-8.72, 1.16]
Leung 2006	60	20	9	50	20	12	1.2%	10.00 [-7.29, 27.29]
Panday 2004a	25.73	15.07	80	45	14	20	2.9%	-19.27 [-26.24, -12.30]
Turan 2003b	7	8	25	11	14	25	3.0%	-4.00 [-10.32, 2.32]
Vahedi 2011	25.8	19.5	36	34	27.2	40	2.1%	-8.20 [-18.77, 2.37]
Özgencil 2011	11	4.8	30	15	7.7	30	3.7%	-4.00 [-7.25, -0.75]
**Subtotal (95% CI)**			**219**			**147**	**16.2%**	**-6.22 [-11.55, -0.89]**
Heterogeneity: Tau² = 29.11; Chi² = 19.84, df = 5 (P = 0.001); I² = 75%								
Test for overall effect: Z = 2.29 (P = 0.02)								
**2.3.6 Thoracic surgery**								
Grosen 2014	6.58	13.61	52	12.43	16.4	52	3.1%	-5.85 [-11.64, -0.06]
Hout 2007	5	59.3	23	0	14.8	28	0.7%	5.00 [-19.85, 29.85]
Kinney 2011	29	18.2	57	31	18.9	68	3.0%	-2.00 [-8.52, 4.52]
Kosucu 2013	13	8	29	32	11	31	3.4%	-19.00 [-23.84, -14.16]
Menda 2010	23	35	30	38	60	30	0.7%	-15.00 [-39.86, 9.86]
Omran 2006	32	38.66	25	54	38.66	25	0.8%	-22.00 [-43.43, -0.57]
Rapchuk 2010	18	33	27	21	34	27	1.1%	-3.00 [-20.87, 14.87]
Ucak 2011	12	0.1	20	30	48	20	0.9%	-18.00 [-39.04, 3.04]
**Subtotal (95% CI)**			**263**			**281**	**13.6%**	**-9.74 [-17.12, -2.36]**
Heterogeneity: Tau² = 60.63; Chi² = 24.70, df = 7 (P = 0.0009); I² = 72%								
Test for overall effect: Z = 2.59 (P = 0.010)								
**Total (95% CI)**			**1340**			**1207**	**100.0%**	**-7.51 [-9.73, -5.30]**
Heterogeneity: Tau² = 32.02; Chi² = 360.05, df = 37 (P < 0.00001); I² = 90%								
Test for overall effect: Z = 6.64 (P < 0.00001)								
Test for subgroup differences: Chi² = 20.30, df = 5 (P = 0.001), I² = 75.4%								

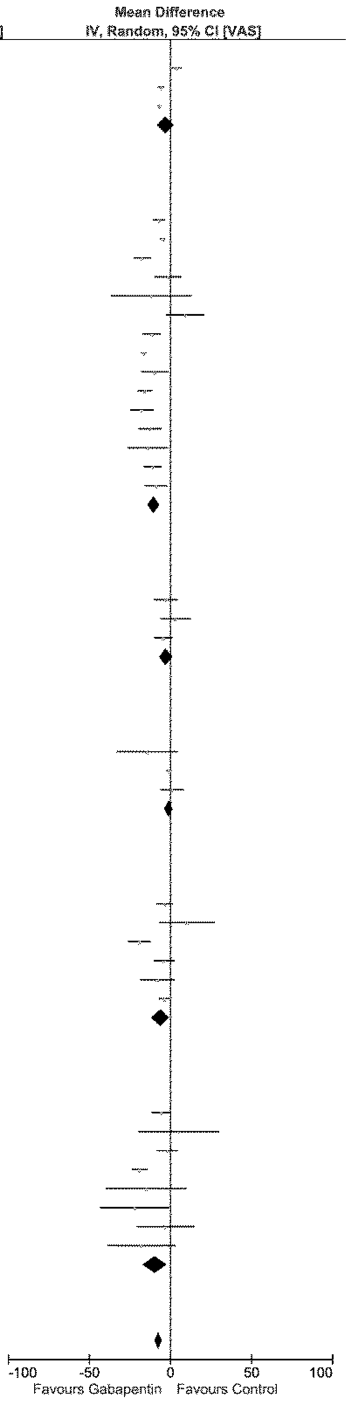

Appendix 15: Forest plot of VAS 24 hours at mobilization, all trials

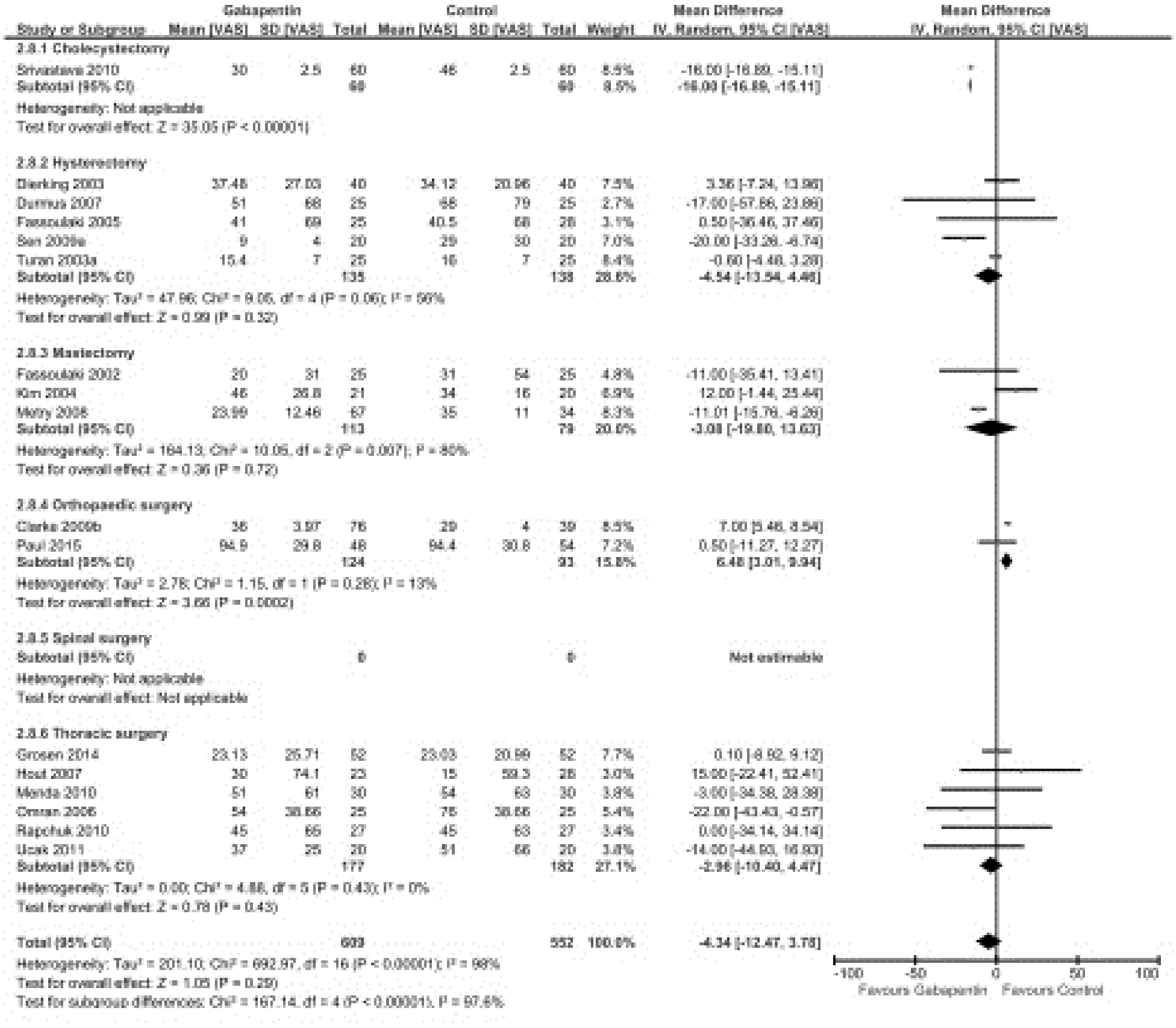

Appendix 16: Forest plot nausea, all trials

Study or Subgroup	Gabapentin Events	Gabapentin Total	Control Events	Control Total	Weight	Risk Ratio M-H, Fixed, 95% CI
**2.5.1 Cholecystectomy**						
Takmaz 2007	9	30	9	15	3.5%	0.50 [0.25, 0.99]
**Subtotal (95% CI)**		**30**		**15**	**3.5%**	**0.50 [0.25, 0.99]**
Total events	9		9			
Heterogeneity: Not applicable						
Test for overall effect: Z = 1.98 (P = 0.05)						
**2.5.2 Hysterectomy**						
Ajori 2011	8	69	19	69	5.6%	0.42 [0.20, 0.90]
Behdad 2012	5	30	5	31	1.4%	1.03 [0.33, 3.21]
Dierking 2003	12	39	11	32	3.5%	0.90 [0.46, 1.75]
Durmus 2007	7	25	9	25	2.6%	0.78 [0.34, 1.76]
Fassoulaki 2006	1	27	5	24	1.6%	0.18 [0.02, 1.42]
Ghafari 2009	5	33	7	33	2.1%	0.71 [0.25, 2.02]
Gilron 2004	4	20	12	22	3.4%	0.37 [0.14, 0.95]
Sekhavet 2009	32	49	37	49	10.9%	0.86 [0.67, 1.12]
Sen 2009a	9	20	8	20	2.3%	1.13 [0.55, 2.32]
Turan 2003a	5	25	7	25	2.1%	0.71 [0.26, 1.95]
Turan 2006	6	25	2	25	0.6%	3.00 [0.67, 13.46]
Verma 2008	5	25	4	25	1.2%	1.25 [0.38, 4.12]
**Subtotal (95% CI)**		**387**		**380**	**37.2%**	**0.77 [0.63, 0.95]**
Total events	99		126			
Heterogeneity: Chi² = 12.74, df = 11 (P = 0.31); I² = 14%						
Test for overall effect: Z = 2.43 (P = 0.01)						
**2.5.3 Mastectomy**						
Doha 2010	2	30	4	29	1.2%	0.48 [0.10, 2.44]
Grover 2009	12	15	6	21	1.5%	2.80 [1.36, 5.76]
Kim 2004	8	21	8	20	2.4%	0.95 [0.44, 2.05]
**Subtotal (95% CI)**		**66**		**70**	**5.1%**	**1.38 [0.85, 2.23]**
Total events	22		18			
Heterogeneity: Chi² = 6.21, df = 2 (P = 0.04); I² = 68%						
Test for overall effect: Z = 1.30 (P = 0.19)						
**2.5.4 Orthopaedic surgery**						
Clarke 2009b	24	76	14	38	5.5%	0.86 [0.50, 1.46]
Clarke 2014	26	88	26	77	8.1%	0.88 [0.56, 1.37]
Paul 2013	33	52	40	49	12.1%	0.78 [0.61, 0.99]
Paul 2015	0	0	0	0		Not estimable
**Subtotal (95% CI)**		**216**		**164**	**25.7%**	**0.83 [0.66, 1.03]**
Total events	83		80			
Heterogeneity: Chi² = 0.31, df = 2 (P = 0.86); I² = 0%						
Test for overall effect: Z = 1.73 (P = 0.08)						
**2.5.5 Spinal surgery**						
Erten 2010	0	39	0	20		Not estimable
Khan 2010	12	150	2	25	1.0%	1.00 [0.24, 4.20]
Khurana 2013	2	30	0	30	0.1%	5.00 [0.25, 99.95]
Panday 2004a	4	80	1	20	0.5%	1.00 [0.12, 8.46]
Panday 2004c	5	28	4	28	1.2%	1.25 [0.37, 4.17]
Radhakrishnan 2005	6	30	6	30	1.8%	1.00 [0.36, 2.75]
Turan 2003b	5	25	7	25	2.1%	0.71 [0.26, 1.95]
Özgencil 2011	8	30	7	30	2.1%	1.14 [0.47, 2.75]
**Subtotal (95% CI)**		**412**		**208**	**8.7%**	**1.07 [0.68, 1.68]**
Total events	42		27			
Heterogeneity: Chi² = 1.75, df = 6 (P = 0.94); I² = 0%						
Test for overall effect: Z = 0.28 (P = 0.78)						
**2.5.6 Thoracic surgery**						
Grosen 2014	25	52	37	52	10.9%	0.68 [0.49, 0.94]
Kosucu 2013	2	29	4	31	1.1%	0.53 [0.11, 2.70]
Menda 2010	9	30	18	30	5.3%	0.50 [0.27, 0.93]
Omran 2006	5	50	7	50	2.1%	0.71 [0.24, 2.10]
Ucak 2011	4	20	2	20	0.6%	2.00 [0.41, 9.71]
**Subtotal (95% CI)**		**181**		**183**	**19.9%**	**0.66 [0.50, 0.88]**
Total events	45		68			
Heterogeneity: Chi² = 2.77, df = 4 (P = 0.60); I² = 0%						
Test for overall effect: Z = 2.83 (P = 0.005)						
**Total (95% CI)**		**1292**		**1020**	**100.0%**	**0.81 [0.72, 0.92]**
Total events	300		328			
Heterogeneity: Chi² = 34.39, df = 30 (P = 0.27); I² = 13%						
Test for overall effect: Z = 3.37 (P = 0.0008)						
Test for subgroup differences: Chi² = 10.10, df = 5 (P = 0.07), I² = 50.5%						

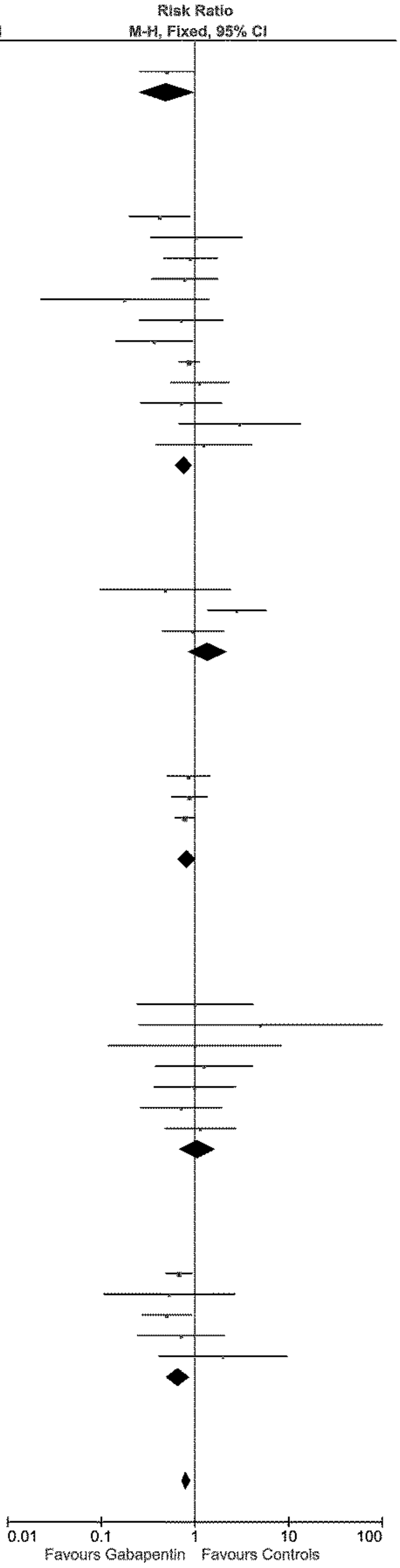

Appendix 17: Forest plot vomiting, all trials

Study or Subgroup	Gabapentin Events	Gabapentin Total	Control Events	Control Total	Weight	Risk Ratio M-H, Fixed, 95% CI
**2.6.1 Cholecystectomy**						
Takmaz 2007	7	30	7	15	4.3%	0.50 [0.21, 1.16]
**Subtotal (95% CI)**		**30**		**15**	**4.3%**	**0.50 [0.21, 1.16]**
Total events	7		7			
Heterogeneity: Not applicable						
Test for overall effect: Z = 1.61 (P = 0.11)						
**2.6.2 Hysterectomy**						
Ajori 2011	5	69	16	69	7.4%	0.31 [0.12, 0.81]
Behdad 2012	5	30	7	31	3.2%	0.74 [0.26, 2.07]
Dierking 2003	18	39	15	32	7.6%	0.98 [0.60, 1.62]
Durmus 2007	3	25	6	25	2.8%	0.50 [0.14, 1.78]
Ghafari 2009	4	33	9	33	4.2%	0.44 [0.15, 1.30]
Sekhavet 2009	18	49	19	49	8.8%	0.95 [0.57, 1.58]
Sen 2009a	8	20	8	20	3.7%	1.00 [0.47, 2.14]
Turan 2003a	6	25	9	25	4.2%	0.67 [0.28, 1.59]
Turan 2006	12	25	18	25	8.3%	0.67 [0.41, 1.07]
Verma 2008	3	25	4	25	1.9%	0.75 [0.19, 3.01]
**Subtotal (95% CI)**		**340**		**334**	**52.0%**	**0.71 [0.57, 0.90]**
Total events	82		111			
Heterogeneity: Chi² = 7.60, df = 9 (P = 0.57); I² = 0%						
Test for overall effect: Z = 2.84 (P = 0.005)						
**2.6.3 Mastectomy**						
Bharti 2012	6	40	9	40	4.2%	0.67 [0.26, 1.70]
Doha 2010	3	30	5	29	2.4%	0.58 [0.15, 2.21]
Grover 2009	7	25	7	21	3.5%	0.84 [0.35, 2.01]
Kim 2004	4	21	2	20	0.9%	1.90 [0.39, 9.28]
Metry 2008	2	77	1	34	0.6%	0.88 [0.08, 9.41]
**Subtotal (95% CI)**		**193**		**144**	**11.6%**	**0.81 [0.48, 1.37]**
Total events	22		24			
Heterogeneity: Chi² = 1.54, df = 4 (P = 0.82); I² = 0%						
Test for overall effect: Z = 0.77 (P = 0.44)						
**2.6.4 Orthopaedic surgery**						
Clarke 2009b	9	76	7	38	4.3%	0.64 [0.26, 1.59]
**Subtotal (95% CI)**		**76**		**38**	**4.3%**	**0.64 [0.26, 1.59]**
Total events	9		7			
Heterogeneity: Not applicable						
Test for overall effect: Z = 0.95 (P = 0.34)						
**2.6.5 Spinal surgery**						
Khan 2010	8	150	1	25	0.8%	1.33 [0.17, 10.21]
Khurana 2013	1	30	2	30	0.9%	0.50 [0.05, 5.22]
Panday 2004a	7	80	2	20	1.5%	0.88 [0.20, 3.89]
Panday 2004c	3	28	4	28	1.9%	0.75 [0.18, 3.05]
Radhakrishnan 2005	2	30	3	30	1.4%	0.67 [0.12, 3.71]
Turan 2003b	1	25	6	25	2.8%	0.17 [0.02, 1.29]
Özgencil 2011	3	50	5	50	2.3%	0.60 [0.15, 2.38]
**Subtotal (95% CI)**		**393**		**208**	**11.5%**	**0.61 [0.33, 1.12]**
Total events	25		23			
Heterogeneity: Chi² = 2.47, df = 6 (P = 0.87); I² = 0%						
Test for overall effect: Z = 1.60 (P = 0.11)						
**2.6.6 Thoracic surgery**						
Grosen 2014	23	52	22	52	10.2%	1.05 [0.67, 1.62]
Kosucu 2013	7	29	4	31	1.8%	1.87 [0.61, 5.73]
Menda 2010	0	30	0	30		Not estimable
Omran 2006	1	50	7	50	3.2%	0.14 [0.02, 1.12]
Ucak 2011	4	20	2	20	0.9%	2.00 [0.41, 9.71]
**Subtotal (95% CI)**		**181**		**183**	**16.1%**	**1.01 [0.69, 1.49]**
Total events	35		35			
Heterogeneity: Chi² = 5.37, df = 3 (P = 0.15); I² = 44%						
Test for overall effect: Z = 0.05 (P = 0.96)						
**Total (95% CI)**		**1213**		**922**	**100.0%**	**0.75 [0.63, 0.89]**
Total events	180		207			
Heterogeneity: Chi² = 21.40, df = 27 (P = 0.77); I² = 0%						
Test for overall effect: Z = 3.30 (P = 0.0010)						
Test for subgroup differences: Chi² = 4.02, df = 5 (P = 0.55), I² = 0%						

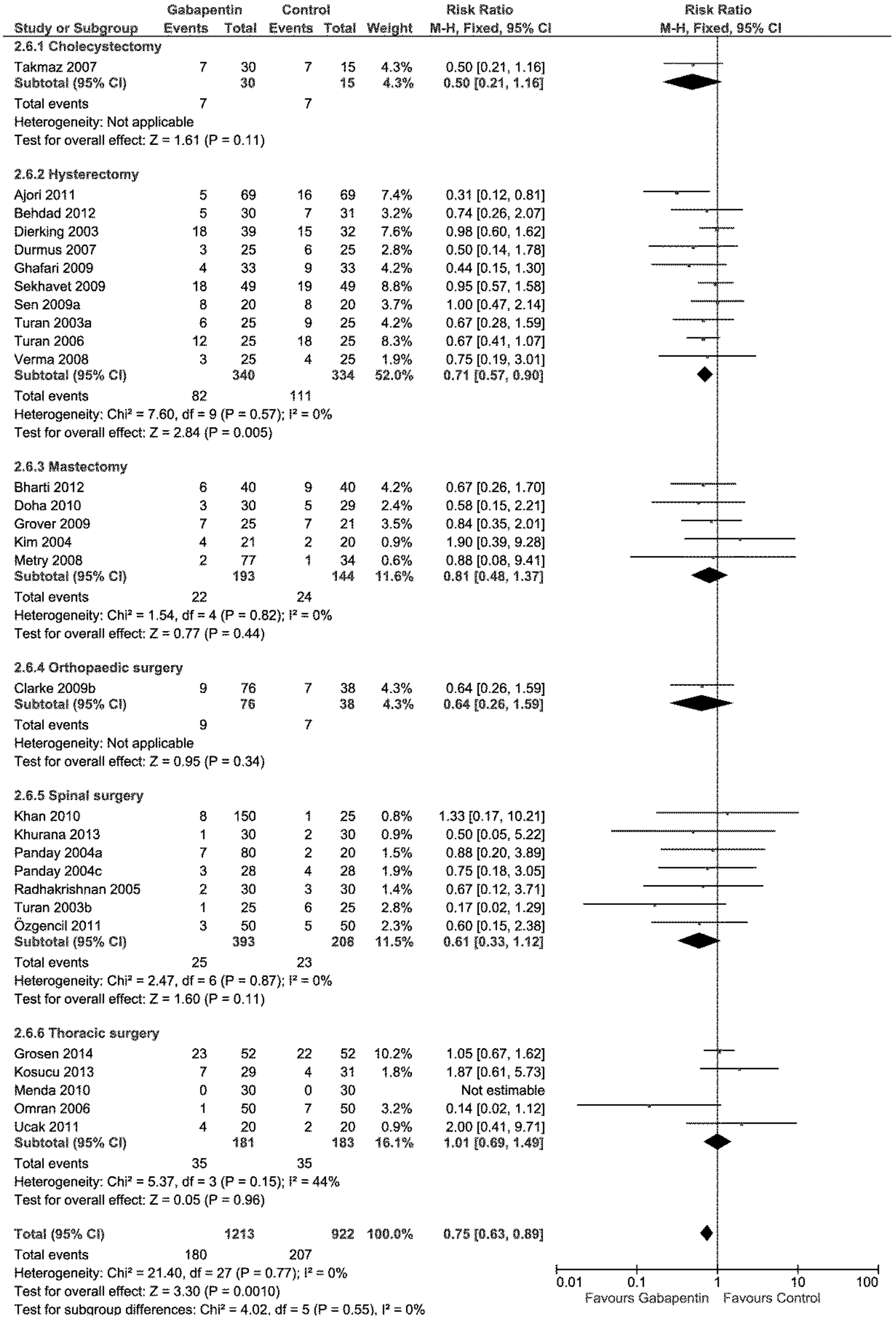

Appendix 18: Forest plot sedation, all trials

Study or Subgroup	Gabapentin Events	Gabapentin Total	Control Events	Control Total	Weight	Risk Ratio M-H, Random, 95% CI
**2.12.1 Cholecystectomy**						
Mishra 2016	12	30	4	30	4.4%	3.00 [1.09, 8.25]
Neogi 2012	1	30	0	30	0.7%	3.00 [0.13, 70.83]
Panday 2004b	52	153	5	153	5.1%	10.40 [4.27, 25.32]
Panday 2006	4	125	2	125	2.2%	2.00 [0.37, 10.72]
Srivastava 2010	14	60	8	60	5.7%	1.75 [0.79, 3.86]
Takmaz 2007	5	30	1	15	1.6%	2.50 [0.32, 19.53]
**Subtotal (95% CI)**		**428**		**413**	**19.8%**	**3.28 [1.55, 6.94]**
Total events	88		20			
Heterogeneity: Tau² = 0.39; Chi² = 10.09, df = 5 (P = 0.07); I² = 50%						
Test for overall effect: Z = 3.11 (P = 0.002)						
**2.12.2 Hysterectomy**						
Ajori 2011	0	69	0	69		Not estimable
Ghafari 2009	2	33	3	33	2.1%	0.67 [0.12, 3.73]
Ghai 2011	10	30	12	30	6.5%	0.83 [0.43, 1.63]
Ray 2015	0	30	0	30		Not estimable
Rorarius 2004	14	38	12	37	6.8%	1.14 [0.61, 2.12]
Sekhavet 2009	26	49	22	49	8.4%	1.18 [0.79, 1.78]
Sen 2009a	3	20	3	20	2.7%	1.00 [0.23, 4.37]
Turan 2003a	1	25	0	25	0.8%	3.00 [0.13, 70.30]
**Subtotal (95% CI)**		**294**		**293**	**27.3%**	**1.08 [0.81, 1.45]**
Total events	56		52			
Heterogeneity: Tau² = 0.00; Chi² = 1.51, df = 5 (P = 0.91); I² = 0%						
Test for overall effect: Z = 0.52 (P = 0.61)						
**2.12.3 Mastectomy**						
Dirks 2002	21	31	22	34	8.8%	1.05 [0.74, 1.48]
Kim 2004	5	21	5	20	4.1%	0.95 [0.32, 2.80]
**Subtotal (95% CI)**		**52**		**54**	**12.9%**	**1.04 [0.75, 1.44]**
Total events	26		27			
Heterogeneity: Tau² = 0.00; Chi² = 0.03, df = 1 (P = 0.86); I² = 0%						
Test for overall effect: Z = 0.22 (P = 0.83)						
**2.12.4 Orthopaedic surgery**						
Clarke 2009b	18	76	7	38	5.7%	1.29 [0.59, 2.81]
Paul 2013	35	52	35	49	9.4%	0.94 [0.73, 1.22]
**Subtotal (95% CI)**		**128**		**87**	**15.1%**	**0.97 [0.76, 1.24]**
Total events	53		42			
Heterogeneity: Tau² = 0.00; Chi² = 0.66, df = 1 (P = 0.42); I² = 0%						
Test for overall effect: Z = 0.23 (P = 0.82)						
**2.12.5 Spinal surgery**						
Erten 2010	0	39	0	20		Not estimable
Khan 2010	8	125	1	25	1.6%	1.60 [0.21, 12.23]
Khurana 2013	4	30	0	30	0.9%	9.00 [0.51, 160.17]
Panday 2004a	0	80	0	20		Not estimable
Radhakrishnan 2005	1	30	1	30	1.0%	1.00 [0.07, 15.26]
Turan 2003b	2	25	1	25	1.3%	2.00 [0.19, 20.67]
Vahedi 2011	16	36	0	40	0.9%	36.57 [2.27, 588.35]
Özgencil 2011	8	30	5	30	4.5%	1.60 [0.59, 4.33]
**Subtotal (95% CI)**		**395**		**220**	**10.3%**	**2.65 [0.94, 7.52]**
Total events	39		8			
Heterogeneity: Tau² = 0.54; Chi² = 7.47, df = 5 (P = 0.19); I² = 33%						
Test for overall effect: Z = 1.83 (P = 0.07)						
**2.12.6 Thoracic surgery**						
Grosen 2014	10	52	7	52	5.1%	1.43 [0.59, 3.47]
Kinney 2011	10	57	10	63	5.6%	1.11 [0.50, 2.46]
Kosucu 2013	2	29	1	31	1.3%	2.14 [0.20, 22.34]
Omran 2006	2	50	1	50	1.3%	2.00 [0.19, 21.36]
Ucak 2011	2	20	1	20	1.3%	2.00 [0.20, 20.33]
**Subtotal (95% CI)**		**208**		**216**	**14.6%**	**1.34 [0.78, 2.32]**
Total events	26		20			
Heterogeneity: Tau² = 0.00; Chi² = 0.62, df = 4 (P = 0.96); I² = 0%						
Test for overall effect: Z = 1.07 (P = 0.29)						
**Total (95% CI)**		**1505**		**1283**	**100.0%**	**1.50 [1.13, 1.99]**
Total events	288		169			
Heterogeneity: Tau² = 0.21; Chi² = 57.56, df = 26 (P = 0.0004); I² = 55%						
Test for overall effect: Z = 2.77 (P = 0.006)						
Test for subgroup differences: Chi² = 12.54, df = 5 (P = 0.03), I² = 60.1%						

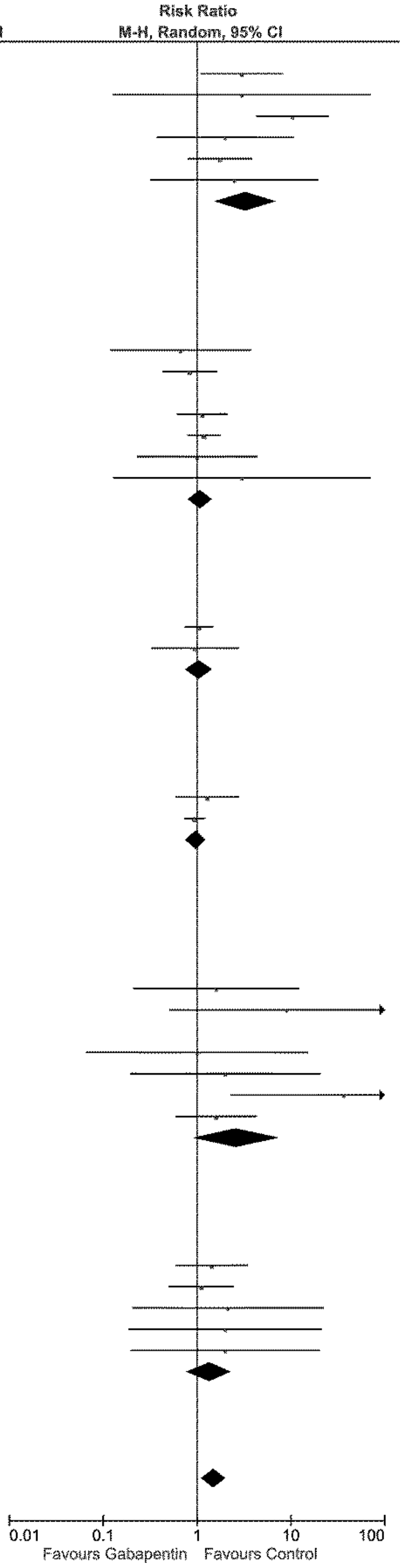

Appendix 19: Forest plot dizziness, all trials

Study or Subgroup	Gabapentin Events	Gabapentin Total	Control Events	Control Total	Weight	Risk Ratio M-H, Random, 95% CI
**2.13.1 Cholecystectomy**						
Bekawi 2014	17	30	25	30	11.8%	0.68 [0.48, 0.97]
Mishra 2016	3	30	6	30	0.9%	0.50 [0.14, 1.82]
Neogi 2012	1	30	1	30	0.2%	1.00 [0.07, 15.26]
Panday 2004b	0	153	0	153		Not estimable
Srivastava 2010	5	60	7	60	1.2%	0.71 [0.24, 2.13]
Takmaz 2007	12	30	7	15	3.0%	0.86 [0.43, 1.72]
**Subtotal (95% CI)**		**333**		**318**	**17.1%**	**0.70 [0.52, 0.94]**
Total events	38		46			
Heterogeneity: Tau² = 0.00; Chi² = 0.68, df = 4 (P = 0.95); I² = 0%						
Test for overall effect: Z = 2.37 (P = 0.02)						
**2.13.2 Hysterectomy**						
Ajori 2011	0	69	0	69		Not estimable
Behdad 2012	0	30	0	31		Not estimable
Dierking 2003	23	39	15	32	7.1%	1.26 [0.80, 1.98]
Fassoulaki 2005	1	25	0	28	0.1%	3.35 [0.14, 78.60]
Ghafari 2009	2	33	2	33	0.4%	1.00 [0.15, 6.68]
Ghai 2011	8	30	1	30	0.4%	8.00 [1.07, 60.09]
Ray 2015	2	30	2	30	0.4%	1.00 [0.15, 6.64]
Rorarius 2004	6	38	4	37	1.0%	1.46 [0.45, 4.76]
Sekhavet 2009	5	49	7	49	1.3%	0.71 [0.24, 2.10]
Sen 2009a	2	20	2	20	0.4%	1.00 [0.16, 6.42]
Turan 2003a	2	25	1	25	0.3%	2.00 [0.19, 20.67]
Turan 2006	6	25	2	25	0.6%	3.00 [0.67, 13.46]
Verma 2008	1	25	0	25	0.1%	3.00 [0.13, 70.30]
**Subtotal (95% CI)**		**438**		**434**	**12.2%**	**1.34 [0.95, 1.89]**
Total events	58		36			
Heterogeneity: Tau² = 0.00; Chi² = 6.78, df = 10 (P = 0.75); I² = 0%						
Test for overall effect: Z = 1.67 (P = 0.10)						
**2.13.3 Mastectomy**						
Azemati 2013	14	50	14	50	3.7%	1.00 [0.53, 1.87]
Dirks 2002	11	31	14	34	3.8%	0.86 [0.46, 1.60]
Doha 2010	8	30	2	29	0.7%	3.87 [0.90, 16.70]
Grover 2009	10	25	7	21	2.4%	1.20 [0.55, 2.60]
Kim 2004	8	21	9	20	2.7%	0.85 [0.41, 1.76]
**Subtotal (95% CI)**		**157**		**154**	**13.3%**	**1.03 [0.74, 1.43]**
Total events	51		46			
Heterogeneity: Tau² = 0.00; Chi² = 4.01, df = 4 (P = 0.40); I² = 0%						
Test for overall effect: Z = 0.16 (P = 0.88)						
**2.13.4 Orthopaedic surgery**						
Clarke 2009b	19	76	8	38	2.7%	1.19 [0.57, 2.46]
Clarke 2014	2	88	12	77	0.7%	0.15 [0.03, 0.63]
Paul 2013	29	52	28	49	12.4%	0.98 [0.69, 1.37]
**Subtotal (95% CI)**		**216**		**164**	**15.8%**	**0.72 [0.32, 1.66]**
Total events	50		48			
Heterogeneity: Tau² = 0.36; Chi² = 7.37, df = 2 (P = 0.03); I² = 73%						
Test for overall effect: Z = 0.76 (P = 0.45)						
**2.13.5 Spinal surgery**						
Khan 2010	5	125	1	25	0.3%	1.00 [0.12, 8.20]
Leung 2006	0	12	0	9		Not estimable
Panday 2004a	0	80	0	20		Not estimable
Panday 2004c	1	28	0	28	0.1%	3.00 [0.13, 70.64]
Turan 2003b	6	25	4	25	1.1%	1.50 [0.48, 4.68]
Özgencil 2011	9	30	6	30	1.8%	1.50 [0.61, 3.69]
**Subtotal (95% CI)**		**300**		**137**	**3.4%**	**1.49 [0.77, 2.86]**
Total events	21		11			
Heterogeneity: Tau² = 0.00; Chi² = 0.33, df = 3 (P = 0.95); I² = 0%						
Test for overall effect: Z = 1.19 (P = 0.24)						
**2.13.6 Thoracic surgery**						
Grosen 2014	41	52	40	52	34.7%	1.02 [0.84, 1.26]
Kinney 2011	9	57	10	63	2.1%	0.99 [0.44, 2.27]
Omran 2006	6	50	4	50	1.0%	1.50 [0.45, 4.99]
Ucak 2011	2	20	1	20	0.3%	2.00 [0.20, 20.33]
**Subtotal (95% CI)**		**179**		**185**	**38.1%**	**1.04 [0.85, 1.26]**
Total events	58		55			
Heterogeneity: Tau² = 0.00; Chi² = 0.80, df = 3 (P = 0.85); I² = 0%						
Test for overall effect: Z = 0.38 (P = 0.70)						
**Total (95% CI)**		**1623**		**1392**	**100.0%**	**1.00 [0.88, 1.12]**
Total events	276		242			
Heterogeneity: Tau² = 0.00; Chi² = 29.26, df = 31 (P = 0.56); I² = 0%						
Test for overall effect: Z = 0.07 (P = 0.94)						
Test for subgroup differences: Chi² = 10.54, df = 5 (P = 0.06), I² = 52.6%						

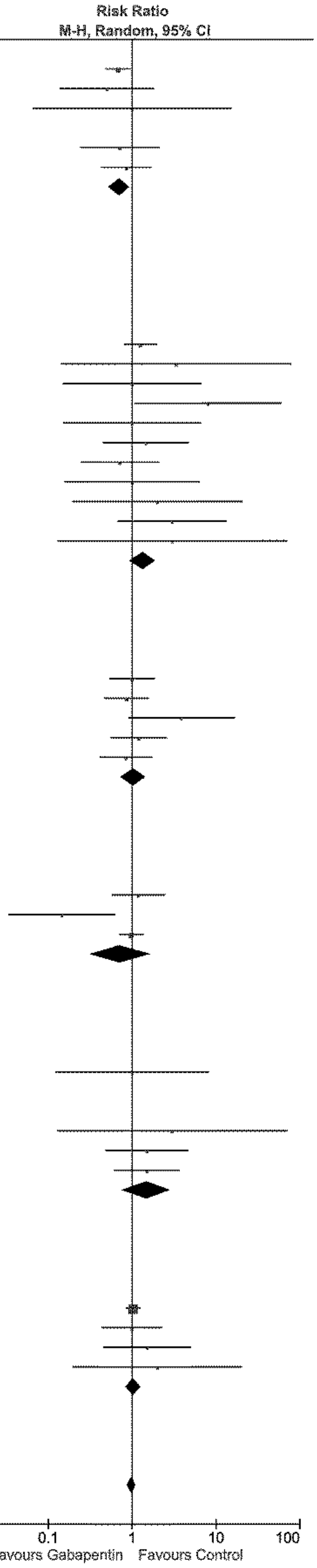

Supplement: Supplementary file 1 — Appendix 1. Search strategies. Appendix 2. Opioid conversion table. Appendix 3. Forest plot of 24-h morphine consumption from all trials estimates. Appendix 4. TSA of 24-h morphine consumption in the cholecystectomy subgroup, all trials. Appendix 5. TSA of 24-h morphine consumption in the hysterectomy subgroup, all trials. Appendix 6. TSA of 24-h morphine consumption in the mastectomy subgroup, all trials. Appendix 7. TSA of 24-h morphine consumption in the orthopedic arthroplasty subgroup, all trials. Appendix 8. TSA of 24-h morphine consumption in the spinal subgroup, all trials. Appendix 9. TSA of 24-h morphine consumption in the thoracic surgery subgroup, all trials. Appendix 10. Forest plot of SAEs, all trials. Appendix 11. TSA of SAEs in the thoracic surgery subgroup, all trials. Appendix 12. Forest plot of VAS 6 h at rest, all trials. Appendix 13. Forest plot of VAS 6 h at mobilization, all trials. Appendix 14. Forest plot of VAS 24 h at rest, all trials. Appendix 15. Forest plot of VAS 24 h at mobilization, all trials. Appendix 16. Forest plot nausea, all trials. Appendix 17. Forest plot vomiting, all trials. Appendix 18. Forest plot sedation, all trials. Appendix 19. Forest plot dizziness, all trials. (PDF 736 kb) [file 12871_2017_373_MOESM1_ESM.pdf]
